# Contribution of 5-HT_2_ Receptors to the Control of the Spinal Locomotor System in Intact Rats

**DOI:** 10.3389/fncir.2020.00014

**Published:** 2020-04-24

**Authors:** Henryk Majczyński, Anna M. Cabaj, Larry M. Jordan, Urszula Sławińska

**Affiliations:** ^1^Nencki Institute of Experimental Biology, Polish Academy of Sciences, Warsaw, Poland; ^2^Department of Physiology and Pathophysiology, University of Manitoba, Winnipeg, MB, Canada

**Keywords:** serotonin, antagonist, inverse agonist, spinal cord, intrathecal application

## Abstract

Applying serotonergic (5-HT) agonists or grafting of fetal serotonergic cells into the spinal cord improves locomotion after spinal cord injury. Little is known about the role of 5-HT receptors in the control of voluntary locomotion, so we administered inverse agonists of 5-HT_2_ (Cyproheptadine; Cypr), 5-HT_2A_ neutral antagonist (Volinanserin; Volin), 5-HT_2C_ neutral antagonist (SB 242084), and 5-HT_2B/2C_ inverse agonist (SB 206553) receptors intrathecally in intact rats and monitored their effects on unrestrained locomotion. An intrathecal cannula was introduced at the low thoracic level and pushed caudally until the tip reached the L2/L3 or L5/L6 spinal segments. Locomotor performance was evaluated using EMG activity of hindlimb muscles during locomotion on a 2 m long runway. Motoneuron excitability was estimated using EMG recordings during dorsi- and plantar flexion at the ankle. Locomotion was dramatically impaired after the blockage of 5-HT_2A_ receptors. The effect of Cypr was more pronounced than that of Volin since in the L5/L6 rats Cypr (but not Volin) induced significant alteration of the strength of interlimb coordination followed by total paralysis. These agents significantly decreased locomotor EMG amplitude and abolished or substantially decreased stretch reflexes. Blocking 5-HT_2B/2C_ receptors had no effect either on locomotion or reflexes. We suggest that in intact rats serotonin controls timing and amplitude of muscle activity by acting on 5-HT_2A_ receptors on both CPG interneurons and motoneurons, while 5-HT_2B/2C_ receptors are not involved in control of the locomotor pattern in lumbar spinal cord.

## Introduction

There is recent evidence that the appearance of locomotor activity after spinal cord injury is due to 5-HT_2C_ receptors that became constitutively active after injury (Fouad et al., 2010; Murray et al., 2010). 5-HT receptors that predominate in the control of locomotion in uninjured animals has not been established, nor has any role for 5-HT_2C_ receptors in normal locomotion investigated. We hypothesized that intrathecal application of antagonists or inverse agonists to the 5-HT receptors implicated in locomotor control in intact rats can provide new insights into the receptors that predominate in voluntary control of locomotion. Previous pharmacological evidence has demonstrated the significant role of serotonergic receptors 5-HT_2A_, 5-HT_2C_, and 5-HT_7_ in the recovery of locomotor function in paraplegic rats.

Pharmacological evidence using agonists has demonstrated contributions of several 5-HT receptors to the initiation and control of locomotion in the isolated spinal cord of neonatal animals, but few studies have tested the contributions of these receptors to the ability of 5-HT to control locomotion in adult, intact animals. Activation of 5-HT_1A_/5-HT_7_ receptors with 8-hydroxy-2-(di-*n*-propylamino)-tetralin (8-OHDPAT) has shown that one or both of these receptors are able to enhance locomotor activity in spinal injured adult rodents (Antri et al., 2003, 2005; Landry et al., 2006; Courtine et al., 2009; Musienko et al., 2011; Sławińska et al., 2012b, 2014a; van den Brand et al., 2012; Cowley et al., 2015) and to control the coordination of flexor and extensor muscles and muscles on the left and right hindlimbs of the animal (Sławińska et al., 2012b, 2014a). Quipazine, a 5-HT_2A_/5-HT_2C_ (but not 5-HT_2B_) agonist, is a potent stimulant of locomotor activity in animals (cats, rats and mice) with spinal cord injury (Barbeau and Rossignol, 1990, 1991; Antri et al., 2002, 2003; Gerasimenko et al., 2007; Courtine et al., 2009; Musienko et al., 2011; Ghosh and Pearse, 2014; Sławińska et al., 2014a). Ung et al. (2008) found that 5-HT_2A_ but not 5-HT_2B_ or 5-HT_2C_ receptors could account for quipazine-induced locomotion in mice 1 week after spinal transection. Here we investigated the role of these receptors in intact adult rats.

Different 5-HT receptors are implicated in the control of separate components of the locomotor rhythm and pattern, such that the use of different specific agonists or antagonists produces different functional consequences (Sławińska et al., 2014a). There is also growing evidence suggesting a regional differentiation of certain receptors in different rostro-caudal regions (Jordan and Schmidt, 2002; Langlet et al., 2005; Liu and Jordan, 2005; Liu et al., 2009) that are in line with the differential effects of 5-HT on supra-lumbar *vs.* lumbar regions. Recent studies have extended this issue to include effects at the sacral level (Murray et al., 2010).

Much of the knowledge about the role(s) of the different receptors has emerged from fictive locomotion experiments *in vitro*, but recent evidence makes it clear that the function and likely targets for specific receptors can change during development, so that the results in neonatal preparations may not reflect the functional role(s) of specific receptors in adult animals. For example, cholinergic agonists applied to the isolated spinal cord of neonates can produce coordinated fictive locomotion (Cowley and Schmidt, 1994; Lev-Tov et al., 2000; Anglister et al., 2008, 2017; Finkel et al., 2014), but when the same agonists are applied to the spinal cord of adult spinal animals they suppressed locomotion, while spinal locomotion was facilitated by cholinergic antagonists (Jordan et al., 2014). In addition, increased and even dominant control by the same cholinergic receptors is exerted on afferent mechanisms controlling locomotion in older animals (Jordan et al., 2014). In such a case the role of specific receptors dramatically changes during development, and the importance of the receptor class in adults cannot be predicted from results on the isolated neonatal spinal cord. Similarly, there is evidence showing that 5-HT receptors undergo developmental changes so that effects of 5-HT on locomotor neurons such as V2a, commissural cells (Husch et al., 2012), and motoneurons (Hounsgaard et al., 1988; Kiehn and Eken, 1998) result in increased excitability in the adult. Evidence has been accumulating for many years that activation of 5-HT receptors can facilitate locomotion in animals with spinal cord injury (Schmidt and Jordan, 2000; Antri et al., 2003, 2005; Sławińska et al., 2014a), and spinal cord injury is associated with changes in receptor density (5-HT_2A_ receptors) and changes in post-translational editing (5-HT_2C_ and perhaps 5-HT_2B_ receptors). Tysseling et al. (2017) confirmed that 5-HT_2C_ receptor constitutive activity is increased after spinal cord contusion, but Hayashi et al. (2010) reported that selective 5-HT_2C_ receptor activation is not effective in improving hindlimb function after incomplete lesions. Kim et al. (2001) revealed that m-chlorophenylpiperazine (*m*-CPP), possessing high efficacy at 5-HT_2C_ receptors, applied systemically, improved motor function in spinal rats with and without grafts of fetal spinal cord. In our preliminary experiments using intrathecal (IT) drug injections in intact rats with indwelling catheters at various levels, we found that the precise placement of the cannula tip was a major factor in producing results on locomotion and on motoneuron output (Sławińska et al., 2012a). In our experiments we test the efficacy of the intrathecal delivery using lidocaine injections. Only those cannula placements where lidocaine impaired locomotion were used in subsequent drug trials. This is a precaution that is neglected in many studies using the IT approach.

The importance of 5-HT_2C_ receptors in the production of hyperexcitability in motoneurons and in the appearance of locomotor activity after spinal cord injury has been emphasized (Fouad et al., 2010; Murray et al., 2010; Tysseling et al., 2017), but the available data about their role in intact animals are sparse. Moreover, in our preceding experiments described above, Cypr could be exerting its effects due to its affinity to other 5-HT receptors, including 5-HT_2B/2C_.

These factors, and the previous suggestion that 5-HT_2C_ receptors are without effect on locomotion in uninjured animals (Fouad et al., 2010), prompted us to examine the action of IT applications of 5-HT antagonists more closely in an attempt to determine the effects of specific antagonists of 5-HT_2_ receptors on locomotion in uninjured rats. Here we use intrathecal application of specific antagonist/inverse agonists to 5-HT_2A_, 5-HT_2B_ and 5-HT_2C_ receptors coupled with EMG recordings during locomotion on a runway to facilitate analysis of effects on coordination and excitatory drive to motoneurons. The EMG recordings gives a unique opportunity to investigate the rhythm of the locomotor pattern by establishing the cycle and burst durations as well as the inter- and intralimb coordination, even if the plantar stepping is deteriorated (Sławińska et al., 2014b). This will allow us to determine the contributions of these receptors to the spinal control of locomotion in adult intact rats.

## Materials and Methods

### Animals

The experiments were carried out on female Wistar rats (*n* = 17), age 3 months at the beginning of experiments and weighing between 250 and 300 g. The animals were kept in individual cages in a room with 12–12 h dark-light cycles. All experiments were conducted with the approval of the First Ethics Committee for Animal Experimentation in Poland and followed EU guidelines on animal care.

### Intrathecal Cannula Implantation

**FIGURE 1:**
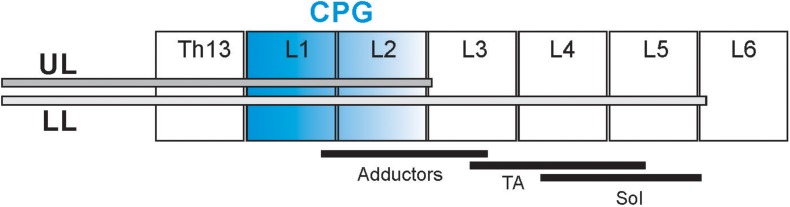
Schematic diagram presenting the cannula tip location in the spinal cord in two groups of rats: upper lumbar (UL) and low lumbar (LL) spinal segments. The black lines below the different spinal cord segments (L2–L5) present schematically the distribution of the motoneurons innervating soleus (Sol), tibialis anterior (TA), and adductor muscles (Add) (Nicolopoulos-Stournaras and Iles, 1983; Sławińska et al., 1995). The segments in blue indicate the hypothetic location of the neural network of the Central Pattern Generators (Cazalets et al., 1995; Jordan and Schmidt, 2002).

The procedure for intrathecal cannula implantation was previously described (Majczyński et al., 2005, 2006; Cabaj et al., 2017). The implantation was performed in aseptic conditions under deep anesthesia (Isoflurane, 2% and Butomidor, 0.05 mg/kg b.w.). A polyethylene cannula (PE-10) was inserted into the subarachnoid space through a small opening made at the Th10/11 vertebral level and was pushed under the dura caudally aiming to reach the L5/L6 (low-lumbar; LL) or L2/L3 (upper-lumbar; UL) root entrance to the spinal cord (Figure 1). The cannula was fixed by sewing it to the Th10 spinous process and stabilized by suturing the overlying back muscles in place. The other end of the cannula was guided under the skin to reach the skull and connected to a custom-made adaptor cemented to the bone. After surgery, the animals received a non-steroidal anti-inflammatory and analgesic treatment (Tolfedine 4 mg/kg s.c.) and antibiotic (Baytril 5 mg/kg s.c.). Two to five days after surgery the patency and correct placement of the cannula was verified by injection of 15 μl of 2% lidocaine followed by 12 μl of sterile saline (sodium chloride 0.9%).

### Implantation of EMG Electrodes

We used EMG (electromyography) recordings to monitor fore- and hindlimb movement to detect the muscles being used for any forward progression observed before and after intrathecal drug application. Bipolar electrodes were implanted in the flexor and/or extensor muscles of each limb [in forelimbs, the *Triceps Brachii* medial head (Tri); in hindlimb muscles: *Adductor Longus* (Add), *Tibialis Anterior* (TA) and *Soleus* (Sol)] under deep anesthesia (Isoflurane, 2 % and Butomidor, 0.05 mg/kg b.w.) (for details see Sławińska et al., 2000; Majczyński et al., 2007). The electrodes were made of Teflon-coated multi-stranded stainless steel wire (0.24 mm in diameter; AS633, Cooner Wire, Chatsworth, CA, United States). The tips of the electrodes with 1–1.5 mm of the insulation removed were pulled through a cutaneous incision on the back of the animal, and each of the hook electrodes was inserted into the appropriate muscle and secured by a suture (Sławińska et al., 2000; Majczyński et al., 2007). The distance between the electrode tips was 1–2 mm. The ground electrode was placed under the skin on the back of the animal some distance from the hindlimb muscles. The connector with the other ends of the wires fixed to it, covered with dental cement (Spofa Dental, Prague, Czechia) and silicone (3140 RTV, Dow Corning), was secured to the back of the animal. After surgery, the animals received a non-steroidal anti-inflammatory and analgesic treatment (Tolfedine, 4 mg/kg s.c.) and antibiotic (Baytril, 5 mg/kg s.c.).

### EMG Recordings and Analysis of Locomotor Pattern on a Horizontal Runway

EMG signals, recorded in freely moving rats, were filtered (0.1- to 1 kHz bandpass), digitized and stored on a computer (2 kHz sampling frequency) using the Winnipeg Spinal Cord Research Centre data capture system. The normal locomotor EMG pattern is characterized by the rhythmic burst activity recorded from the extensor and flexor muscles of the limbs. Based on this rhythmic EMG burst activity various indices to quantify locomotor performance were established using custom software.^1^ First, we selected the fragments of the EMG recordings with 10–14 steps of rhythmic locomotor performance from each rat in different experimental conditions. In the case of a very strong deterioration of locomotor performance, we selected 8–10 of the EMG cycles reflecting impaired hindlimb movement with no plantar stepping. In the case of drug-induced total paralysis, we selected the last trial of locomotor progression along the runway presenting burst EMG activity, prior to the complete loss of EMG that usually appears in 3–5 min after drug application (e.g., the potent effect of Cypr application). The analysis started with marking all the burst onsets and offsets in all the EMG activity recorded. Based on marked burst onsets and offsets we established the cycle duration, burst duration, burst EMG amplitude and inter- and intralimb coordination in different rats for different experimental conditions. The left-right coordination (L-R; the coupling of homonymus muscles of the left and right limb) as well as coordination between flexor and extensor muscles on one side, was determined using polar plot analysis (Batschelet, 1981; Kjaerulff and Kiehn, 1996; Cowley et al., 2005; Zar, 2010; Sławińska et al., 2013). In the polar plot analysis, the position of the vector at 0 or 360° of the polar plot reflects synchrony of analyzed EMG burst onsets, whereas 180° is equivalent to alternation. The length of the vector (*r*, ranging from 0 to 1) indicates the strength of coordination between analyzed muscle burst onsets demonstrating the concentration of phase shift values around the mean. The phase shift values indicated as individual dots on the polar plot should be highly concentrated around the mean phase when the onset of EMG bursts of the same muscle of left and right hindlimbs are strongly coupled. When there is no coupling in analyzed burst muscle onsets, the muscle activities are independent and the distribution of phases should show some dispersion, with the wide distribution of dots on the polar plot. To determine whether the inter- and intralimb coordination *r*-values were concentrated, suggesting coupling of burst activity, or dispersed, indicating no coordination, Rayleigh’s circular statistical test was applied. In our analysis, the inter- or intralimb coordination was considered to be phase-related if *r* was greater than critical Rayleigh’s value (cR) for a given *P*-value (Zar, 2010). When describing these results, we established the *r-*value for each polar plot together with its statistical significance (*p*).

### Testing the Effects of Intrathecal Drug Applications

To block 5-HT_2A_ receptors we used: Cyproheptadine chloride (inverse agonist) [4-(5*H*-Dibenzo[*a*,*d*]cyclohepten-5-ylidine)-methylpiperidine hydrochloride] and Volinanserin hydrochloride salt (selective 5-HT_2A_ antagonist) [(ααR)-α-(2,3-Dimethoxyphenyl)-1-[2-(4-fluorophenyl)ethyl]-4-piperidinemethanol Hydrochloride]. To block 5-HT_2C_ receptors we used SB 242084 (selective 5-HT_2C_ antagonist) (6-Chloro-2,3-dihydro-5-methyl-*N*-[6-[(2-methyl-3-pyridinyl) oxy]-3-pyridinyl]-1*H*-indole-1-carboxyamide dihydrochloride). To block 5-HT_2B_ and 5-HT_2C_ receptors we used SB 206553 hydrochloride (inverse agonist) (3,5-Dihydro-5-methyl-*N*-3-pyridinylbenzo[1,2-*b*:4,5-*b*’]dipyrrole-1(2*H*)-carboxamide hydrochloride. In Table 1 the affinity of agents we employed for different 5-HT receptors is presented.

**TABLE 1:** Chemical agents used for intrathecal application.

**Agent**	**Dose**	**5-HT2A**	**5-HT2B**	**5-HT2C**	**5-HT7**
Cyproheptadine	150 μg in 20 μl	*K*_*i*_ = 0.44 nM	*K*_*i*_ = 1.54 nM	*K*_*i*_ = 2.23 nM	*K*_*i*_ = 7.5 nM^a^
Volinanserin Hydrochloride MDL 100907	300 μg in 30 μl	*K*_*i*_ = 0.36–0.85 nM^b^	*K*_*i*_ = 261 nM	*K*_*i*_ = 88 nM^b^	No data
SB242084	84 μg (6 mM) in 30 μl	*K*_*i*_ = 158.48 nM	*K*_*i*_> 45 nM	*K*_*i*_ = 0.47 nM	*K*_*i*_ = 794.32 nM
SB206553	60 μg (6 mM) in 30 μl	*K*_*i*_ > 1,000 nM	*K*_*i*_ = 1.288 nM	K_*i*_ = 3.16 nM	*K*_*i*_ = 2000 nM

*https://pdsp.unc.edu/databases/pdsp.php. ^*a*^Leopoldo et al., 2011. ^*b*^https://www.caymanchem.com/product/15936 Volinanserin (MDL 100907).*

All the antagonists and inverse agonists were dissolved for stock solution: Cypr and Volin in Glycerol (plus 10 μl 0.1N HCl) as well as SB 206553 and SB 242084 in 30% DMSO. The final dose was prepared from the stock solution by adding 0.9% NaCl just before IT application. None of the described vehicles injected alone in control experiments evoked any changes in locomotion of tested rats.

Intrathecal application of four drugs listed in the Table 1, was performed in 32 independent experiments carried out on 17 rats. Care was taken to use each animal in at least two experiments with two different drugs. The experiments with intrathecal applications were performed usually once a week always separated by an interval of at least 72 h. Such precautions were taken due to the possible effect of tachyphylaxis, which we observed in our previous investigation using the intrathecal application in intact rats (Majczyński et al., 2006). Every intrathecal application of drugs was performed as a single bolus of 20 or 30 μl of dissolved drug in vehicles (for details see below) that was followed by a bolus of about 12 μl of sterile saline to wash the drug from the cannula. The procedure of drug injection lasted approximately 30 s. In the case of no response to the drug tested during a given trial, lidocaine was subsequently injected to test the patency of the cannula. A similar volume of sterile 0.9% saline was used as a control injection.

### Verification of the Cannula Tip Location Within the Spinal Cord

After completing all the experiments each rat was anesthetized and perfused through the heart with PBS followed by 4% paraformaldehyde. The spinal cord was exposed and the level of the entrance of the cannula and the location of its tip was verified by identifying the relevant spinal roots. In some animals, the position of the tip of the cannula was also marked by injecting 20 μl of a dye (gentian violet 1%). It was detected that the dye solution was marking clearly the spinal cord segment close to the tip of cannula but was often detected in the spinal cord along the cannula due to osmotic forces, which drove the dye solution up of the spinal cord along the cannula.

### Locomotor Performance

The locomotion was tested on the horizontal runway 2 m long, 0.12 m wide positioned 1.4 m above the ground with illuminated start platform at one end and a darkened goal box on the other (for details see Majczyński et al., 2007). Rats were trained to pass along the runway with relatively constant velocity and stable rhythmic locomotor movements. Usually, they present 12–14 steps to pass the 2 m long runway. In all the experiments only runs with at least 10 regular steps were accepted for further analysis.

Each experiment started with the evaluation of pre-drug locomotor performance. Then a bolus of 20 or 30 μl of selected antagonist suspension (see Table 1) was delivered through the intrathecal cannula and washed out with 12 μl of saline and locomotion was tested immediately when the first sign of hindlimb movement deterioration (usually 3 – 5 min after drug administration) and then was continued at 15–20 min intervals for up to 2–3 h to investigate drug effects.

### Reflexes

Reflex responses to dorsi- and plantar flexion of the ankle joint were obtained as previously described (Hnik et al., 1981, 1982; Vejsada et al., 1991; Sławińska and Kasicki, 2002; Cabaj et al., 2017) in an attempt to determine whether the 5-HT_2A_ and 5-HT_2B/2C_ receptor ligands have any direct effect on excitability of motoneurons innervating hindlimb muscles.

### Statistics

For standard statistical analysis regarding the comparison of various parameters established in the different groups of animals, we used the GraphPad Software (GraphPad Software version 8.1.1 for Windows, San Diego, CA, United States).^2^ The data described in our paper are always presented in the text as mean ± standard deviation (mean ± SD). For comparison of the cycle and EMG burst durations, ANOVA with Sidak’s test for multiple comparisons was applied. The relationship between burst duration *vs.* cycle duration established for individual muscles was determined using regression line analysis (Halbertsma, 1983; Majczyński et al., 2007). The data expressed as a ratio of pre-drug value are illustrated in the figures using box-and-whisker diagrams. The boxes are bounded by 25 and 75% quartiles. The median is indicated by the horizontal line. The whiskers extending from each end of the boxes show the extent of the range of the data (the min and max of analyzed values). For the data expressed as the ratio of the control values established in the pre-drug conditions, non-parametric Kruskal–Wallis (KW) statistical analysis with Dunn’s test for multiple comparisons was used. The same statistic was used for comparison of the strength of intra- and interlimb coordination established as the length of the *r-*vector in different experimental conditions. The results of KW non-parametric statistical analysis are provided in the text describing results illustrated in the particular figure as KW (degree of freedom) = statistics value and *p*-value.

## Results

### Alteration of Locomotor Performance in Adult Intact Rats by Interfering With 5-HT_2A_, 5-HT_2B_, or 5-HT_2B/2C_ Receptors – General Observations

**FIGURE 2:**
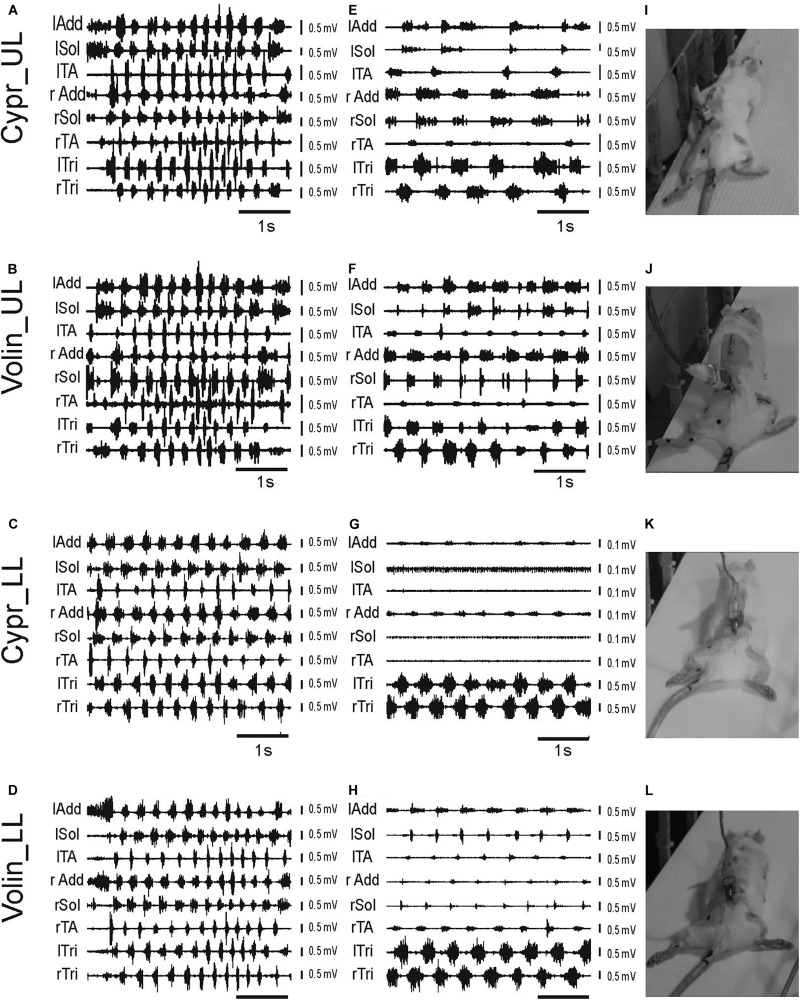
Examples of EMG recordings illustrating hindlimb locomotor impairments in adult freely moving rats induced by blockage of various 5-HT receptors after IT application of either Cyproheptadine (Cypr; **A,E,I,C,G,K**) or Volinanserin (Volin; **B,F,J,D,H,L**) in the upper lumbar (UL) or low lumbar (LL) spinal cord. Left panels **(A–D)** present examples of typical EMG bursts recorded during voluntary locomotion along the horizontal runway in adult freely moving rats in the control pre-drug situation. Middle panels **(E–H)** present examples of EMG activity recorded 3–5 min after the drug administration when usually the highest deterioration of locomotor performance was obtained. Note that 3–5 min after either drug administration **(E–H)** the hindlimb EMG was greatly reduced in amplitude and frequency in all cases. We took care to analyze 8 – 10 rhythmic cycles from the period of highest deterioration (just prior to total paralysis in the case of Cypr treatment in LL rat). All the rats lost hindlimb body weight support and plantar stepping abilities after blockade of 5-HT_2A_ receptors. The increase in EMG amplitude of forelimb muscles (lTri and rTri) reflects the increased use of the forelimbs for progression. See that in LL rats after Cypr application the rhythmic EMG activity of Sol and TA disappeared completely **(G)**, Right panel **(I–L)** presents the individual frames illustrating hindlimb paralysis observed after IT drug applications (see video material presented in Supplementary Information attached to the paper). lAdd/rAdd, left/right adductor muscle; lSol/rSol, left/right soleus muscle; lTA/rTA, left/right tibialis anterior muscle; lTri/rTri, left/right triceps brachii muscle; Cypr, Cyproheptadine; Volin, Volinanserin; UL, upper lumbar segments (L2/L3); LL, low lumbar spinal cord (L5/L6).

Prior to drug administration, the rat locomotor performance along the horizontal runway was tested together with simultaneous recording of EMG activity of selected muscles of fore- and hindlimbs as well as videotaping, as described previously (Cabaj et al., 2017). After completing the video and EMG recordings in the control pre-drug experiments (see the EMG examples in Figures 2A–D) the rats were subjected to the intrathecal (IT) drug application and their locomotor performance was investigated in the same experimental condition, starting at the first sign of drug effect. Application of Cypr or Volin caused the rat to lose body weight support (BWS) in the hindlimbs (see the videos in Supplementary Videos S1–S5). The rats moved forward along the runway using their forelimbs (see the changes in EMG activity of left and right Tri in Figures 2E–H) with their hindquarters, trunk and hindlimbs dragging behind (Figures 2I–L).

After Volin, in both UL (*n* = 5) and LL (*n* = 5) rats, small rhythmic movements at the hip, knee and ankle joints were observed despite the lack of BWS (Figures 2J,L). This was accompanied by rhythmic EMG activity in hindlimb muscles (Figures 2F,H), although the EMG amplitude was smaller than before drug application (Figures 2B,D). After this stage of impairment, which lasted 10–20 min, the BWS gradually returned to the pre-drug level and animals started to use their hindlimbs to perform regular four-limb locomotion. Full recovery appeared after 30–40 min.

After Cypr in the UL (*n* = 6) rats the time course of hindlimb locomotor deterioration was similar to that after Volin, but in LL (*n* = 5) rats the deterioration was more pronounced. In four out of five of LL rats, Cypr evoked total paralysis with a complete absence of EMG activity in the TA and Sol muscles with rather little residual activity present in the Add muscles (Figure 2G). At this stage, which lasted 5–15 min, no movement at any of the hindlimb joints was observed. The legs remained passively extended behind the body and the rats moved forward using their forelimbs only (see EMG recordings in the left and right Tri muscles Figure 2G). After a period of complete hindlimb paralysis induced by the Cypr, small movements in all three joints occurred, and the sequence of recovery was similar to that observed in both groups of rats after Volin. The recovery of four-limb locomotion appeared 40–60 min after drug administration.

Next, we investigated the effect of blockage of the 5HT_2C_ receptors by the neutral antagonist SB 242084 (SB 242; *n* = 6) and of the 5-HT_2B/2C_ receptors by application of the inverse agonist SB 206553 (SB 206; *n* = 5) and we found that they did not evoke any visible changes in locomotor performance of all tested rats up to 1 h after drug applications (see Supplementary Videos S6, S7). Rats moved with similar velocity as pre-drug, and the amplitude of EMG activity in all muscles with implanted electrodes was similar to that before the application of 5HT_2B/2C_ receptor ligands (see EMG examples in Supplementary Figure S1). The effects were similar in LL and UL rats, so the results were pooled.

General observation of EMG activity suggests that 5-HT_2A_ receptors but not 5-HT_2B/2C_ predominated in the voluntary control of locomotion in intact adult rats.

In the following paragraphs, the general observations described above are confirmed using analysis of hindlimb movement indices like the cycle duration, burst duration, amplitude of EMG bursts as well as inter- and intralimb coordination that were established on the basis of EMG burst recordings from rats during locomotor progression along the runway investigated after IT application of different 5-HT receptor antagonists.

### Changes in Locomotor Performance – EMG Activity Analysis

As it was described above the rats after IT application of Cypr or Volin were not able to present any proper hindlimb plantar stepping during progression along the runway. In effect of these drug applications, the rats were losing BWS but in the hindlimb EMG activity still, some rhythmic burst activity could be obtained. Although the appearance of total paralysis was sufficient to illustrate the marked difference between the results of blockage of 5-HT_2A_ receptor and other receptors that have been implicated in recovery of locomotion after spinal cord injury such as 5-HT_2B_ and 5-HT_2C_, we investigated rhythmic movements that persisted just prior to total paralysis and compared to the locomotor EMG pattern induced by the other drug administration that did not result in total paralysis. This gave us the opportunity to analyze the changes that appeared just prior to the paralysis in order to provide an explanation for the loss of weight supported plantar stepping, such as shorten EMG burst duration and reduced amplitude in extensor Sol muscle as well as the alterations of the inter- and intralimb coordination.

### Step Cycle Duration

**FIGURE 3:**
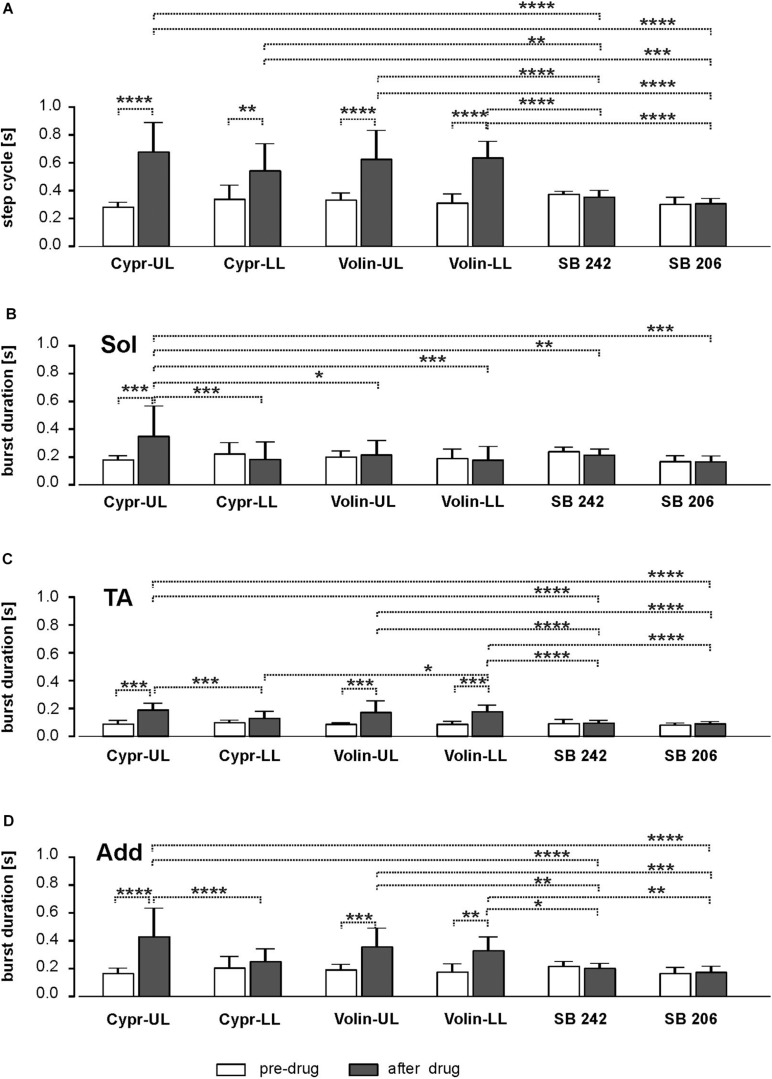
Bar diagram presenting the cycle duration **(A)** and EMG burst duration of Sol **(B)**, TA **(C)** and Add **(D)** muscles before and after blockade of various 5-HT receptors by IT application of Cyproheptadine, Volinanserin, SB 242084 and SB 206553 in the upper lumbar (UL) or low lumbar (LL) spinal cord [mean ± Standard Deviation (SD)]. Results from left and right muscles were pooled in all the rats in the various experimental conditions, so the number of samples taken for analysis were: Cypr_UL *n* = 12, Cypr_LL *n* = 10, Volin_UL *n* = 10, Volin LL *n* = 10, SB 242 *n* = 12, SB 206 *n* = 10. Cypr, Cyproheptadine; Volin, Volinanserin; SB 242, SB 242084; SB 206, SB 206553; UL, upper lumbar spinal cord (L2/L3); LL, low lumbar spinal cord (L5/L6). The significance of the results was tested using ANOVA test followed by Sidak’s test for multiple comparisons (*p < 0.05, **p < 0.01, ***p < 0.001, ****p < 0.0001).

We found that cycle duration, determined as the time between the onset of two consecutive TA EMG bursts was prolonged after Cypr and Volin applications but not after SB 242 or SB 206 where the proper plantar stepping was present without any changes (Figure 3A). Our analysis demonstrated that the changes in cycle duration were significant after Cypr and after Volin application but not after SB 242 or SB 206 [ANOVA; *F*(11,116) = 19.39, *p* < 0.0001]. The *post hoc* analysis (Sidak’s multiple comparisons) indicated that Cypr application significantly prolonged the cycle duration in the UL rats up to 684.2 ± 205.1 ms (pre-drug 286.9 ± 28.9 ms; *p* < 0.0001) and in LL rats up to 540.1 ± 188.3 ms (pre-drug 344.5 ± 95.3 ms; *p* < 0.002) while after Volin the step cycle duration was significantly prolonged in the UL rats up to 630.9 ± 201.8 ms (pre-drug 338.1 ± 44.46 ms; *p* < 0.0001) and in LL rats up to 641.2 ± 113.5 ms (pre-drug 317.5 ± 58.4 ms; *p* < 0.0001). At the same time, the *post hoc* analysis demonstrated that there was no difference in the prolonged cycles after either drug in either group of rats (*p* > 0.05). Moreover, there was no significant difference in step cycle duration after SB 242 or SB 206 applications (*p* > 0.05). In addition, the cycles prolonged by Cypr or Volin applications were significantly different from those unaffected by SB 242 or SB 206. Thus, our results show that Cyproheptadine and Volinanserin applications prolonged the locomotor cycle in the same manner interfering with the 5-HT_2A_ receptors located at UL as well as LL segments while interfering with 5-HT_2B/2C_ receptors did not affect the locomotor performance in adults rats. This step cycle prolongation is likely due to action on the locomotor CPG because actions restricted to motoneurons should not alter step cycle duration. Note that the application of the drugs at the LL site can result in the drug influencing both sites because the drug is very likely to diffuse up along the cannula from the LL to the UL region. Thus LL applications can exert actions on CPG and pattern formation components in addition to having a direct action on motoneurons.

### Burst Duration

Next, we analyzed the changes in the EMG burst duration of the Sol, TA and Add hindlimb muscles after interfering with various 5-HT receptors with IT applications of different ligands (Figures 3B–D).

Our results show that the Sol EMG burst was significantly prolonged only in the Cypr UL (186.2 ± 24.2 *vs.* 356.3 ± 211.2) group of animals [ANOVA; *F*(11,116) = 3.599, *p* = 0.0002; followed by Sidak’s test for multiple comparisons *p* = 0.0002], while it did not change significantly in spite of significant cycle duration prolongation in rats from Cypr LL (228.8 ± 75.6 *vs.* 190.4 ± 119.1), Volin UL (206.7 ± 37.5 *vs.* 222.5 ± 96.5) and Volin LL (196.6 ± 61.5 *vs.* 185.3 ± 91.7) groups (*p* > 0.05). The results in which there is no prolongation of extensor EMG burst duration with prolongation of cycle durations have to be explained with particular caution. It is known that in regular locomotor performance the extensor activity is responsible for the stance phase of the step cycle and the longer extensor muscle activity is related to a longer step cycle (Halbertsma, 1983; Majczyński et al., 2007). Thus, lack of extensor burst prolongation in the case of prolonged cycle duration (see results Figure 3A vs. Figure 3B) indicates crucial deterioration of this muscle activity after interfering with 5-HT receptors by Cypr as well as after Volin IT application. In contrast to the crucial deterioration of EMG activity after Cypr and Volin applications, our results demonstrate that there was no change in Sol EMG burst duration after IT application of SB 242 (*p* > 0.05) and SB 206 (*p* > 0.05). In this case, the lack of significant changes in the burst duration and no change of the step cycle confirms the lack of effects of blockage of 5-HT_2B/2C_ receptors on locomotor performance.

Using the regression line analysis, which is a standard procedure to establish a normal locomotor pattern (Halbertsma, 1983; Majczyński et al., 2007; Frigon and Gossard, 2009), we performed an additional analysis regarding the relationship between EMG burst and step cycle durations. For the regression line analysis, we selected the episodes of rhythmic locomotor activity lasting at least 4 s with 12–14 steps during a good locomotor performance along the runway. In a few cases in which the locomotor performance was dramatically deteriorated after drug applications and animals did not perform plantar stepping, we selected EMG rhythmic activity with at least 8–10 cycles present in the recorded muscles (see examples illustrated after drug applications in Figure 2 middle panels). In the control pre-drug locomotor performance the duration of EMG burst of extensor muscle (soleus or adductor) activity was positively related to step cycle duration, and the regression lines describing their relationship was characterized by a significant slope and a high correlation coefficient (see Supplementary Figures S2A–C). The comparison of the slopes of regression lines revealed that the application of Cypr or Volin evoked significant changes in the relationship between the step cycle duration and the EMG burst duration in the Sol muscles (see changes in the slopes Supplementary Figure S2D). Non-parametric Kruskal–Wallis analysis indicated the significant reduction of the regression line slopes established for the relationship of Sol burst duration *vs.* cycle duration [*K*(11 = 53.90; *p* < 0.0001] in the Cypr LL, Volin UL and Volin LL groups of animals (followed by Dunn’s test for multiple comparisons; *p* < 0.01, *p* < 0.05, *p* < 0.01, respectively). In contrast to the crucial deterioration of Sol EMG burst activity after Cypr and Volin applications, our results demonstrate that there was no change of the regression line slopes after IT application of SB 242 and SB 206 (*p* > 0.05).

Next, we analyzed the EMG burst duration of flexor muscles (tibialis anterior) that remains unchanged with a step cycle duration at different speeds in the normal locomotor performance, and the relationship is described by a flat regression line with a slope close to zero and low correlation coefficient. The analysis of TA EMG activity after Cypr and Volin application (Figure 3C) revealed that the TA burst duration was prolonged in rats from Cypr UL (96.6 ± 17.6 *vs.* 196.3 ± 41.4) as well as from Volin UL (92.7 ± 6.5 *vs.* 179.1 ± 76.5) and Volin LL (93.4 ± 14.68 *vs.* 183.4 ± 41.3) groups [ANOVA; *F*(11,116) = 17.14; *p* < 0.0001; followed by Sidak’s test for multiple comparisons *p* < 0.0001 for all three groups]. Moreover, these TA burst durations were significantly longer (*p* < 0.0001), in comparison to those obtained after SB 242 (98.8 ± 22.8 *vs.* 102.1 ± 12.7) and SB 206 (87.9 ± 8.7 *vs.* 97.27 ± 9.4), which were not affected by interfering with 5-HT_2B/2C_ receptors (*p* > 0.05).

This prolongation of TA EMG burst was not confirmed by the analysis of regression line slopes that revealed that the relationship between the cycle duration and the burst duration of EMG of TA muscles did not change after Cypr as well as after Volin application in UL and LL rats [*K*(11) = 25.83; *p* < 0.01; followed by Dunn’s multiple comparisons *p* > 0.05 for all groups; Supplementary Figures S3A–D). There was no significant change of the regression line slopes for the TA EMG burst *vs.* step cycles after IT application of SB 242 and SB 206 (*p* > 0.05).

In contrast to Sol extensor muscle, the Add EMG burst (hip extensor) was significantly prolonged (Figure 3D) in rats from Cypr UL (170.2 ± 33.27 *vs.* 435.2 ± 199.5), Volin UL (197.2 ± 33.2 *vs.* 361.7 ± 128.3) and Volin LL (182.2 ± 52.1 *vs.* 334.7 ± 92.5) groups [ANOVA; *F*(11,116) = 11.00, *p* < 0.0001; followed by Sidak’s test for multiple comparisons; *p <* 0.0001; *p <* 0.001; *p <* 0.005, respectively], but not in rats from Cypr LL group (*p >* 0.05). The additional analysis of regression line slopes confirmed that the relationship between the cycle duration and the burst duration of EMG of Add muscles did not change after Cypr as well as after Volin application in UL and LL rats (see Supplementary Figures S4A–D). Our results demonstrate also that there was no change of the regression line slopes for the Add EMG burst *vs*. cycles after IT application of SB 242 and SB 206 (*p* > 0.05).

Our results indicate that the blockade of 5-HT_2A_ receptors prolonged the step cycle duration. However, the longer step cycle duration was not related to prolonged extensor Sol muscle burst activity as it is in the control locomotion. The absence of prolongation of the extensor burst duration together with the prolonged cycle duration resulted in the inability of the paralyzed hindlimbs to support the body weight; the hindlimbs were abducted with the burst duration of Add muscle remaining related to the cycle. The hindlimb postural muscles were ineffective for the production of plantar stepping (see Figure 2). Thus, the results of the 5-HT_2A_ receptor blockade on locomotor performance of intact adult rats strongly depend on the placement of the cannula tip in the lumbar spinal cord segments (UL *vs.* LL). The absence of prolonged burst duration of Sol muscle with prolonged cycle duration provides an explanation for the loss of weight support.

### EMG Amplitude

Another factor that might contribute to the abducted posture and the absence of plantar stepping that occurs after 5-HT_2A_ blockade is the force that is produced by the muscle contraction, a feature reflected in the amplitude of the EMG.

**FIGURE 4:**
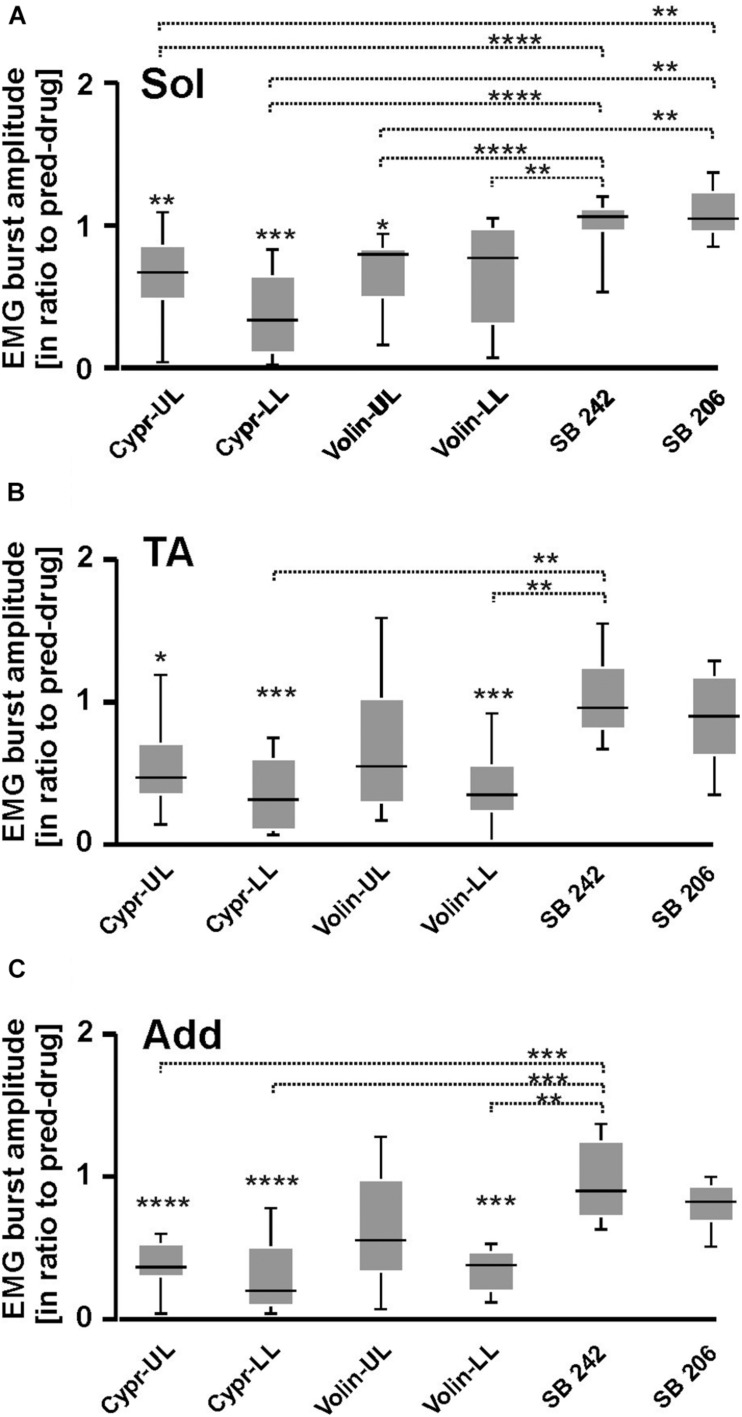
EMG amplitude of soleus **(A)**, tibialis anterior **(B),** and adductor **(C)** muscles resulting from 5-HT receptor blockage by either Cyproheptadine, Volinanserin or SB 242084 or SB 206553 application in the lumbar spinal cord established for muscles of both rat hindlimbs. The significance of the results was tested using non-parametric Kruskal–Wallis test followed by Dunn’s multiple comparisons (*p < 0.05, **p < 0.01, ***p < 0.001, ****p < 0.0001). Results from left and right muscles were pooled in all the rats in the various experimental conditions, so the number of samples taken for analysis were: Cypr_UL *n* = 12, Cypr_LL *n* = 10, Volin_UL *n* = 10, Volin LL *n* = 10, SB 242 *n* = 12, SB 206 *n* = 10. Add, adductor muscle; Sol, soleus muscle; TA, tibialis anterior muscle; Cypr, Cyproheptadine; Volin, Volinanserin; SB 242, SB 242084; SB 206, SB 206553; UL, upper lumbar segments (L2/L3); LL, low lumbar (L5/L6) segments.

Our analysis demonstrated that the EMG burst amplitude during voluntary locomotion on a runway was significantly altered after intrathecal application of Cypr and Volin in Sol [KW(11) = 76.75, *p* < 0.0001], TA [KW(11) = 65.56, *p* < 0.0001] and Add [KW(11) = 90.64, *p* < 0.0001] muscles of both hindlimbs (Figure 4), but not after SB 242 and SB 206 applications (Dunn’s test for multiple comparisons *p* > 0.05 for both groups). EMG burst amplitude before drug application was considered as 1. After Cypr application, Sol EMG burst amplitude was reduced to 0.64 ± 0.29 (*p <* 0.01) in UL rats and to 0.38 ± 0.29 (*p* < 0.001) in LL rats. In contrast, Volin application (Figure 4A) induced a reduction of amplitude to 0.68 ± 0.26 only in UL rats (*p* < 0.01). The TA EMG burst amplitude was reduced after Cypr to 0.55 ± 0.33 (*p* < 0.05) in UL rats and to 0.36 ± 0.26 (*p* < 0.001) in LL rats (Figure 4B). After Volin application, the amplitude significantly changed only in the LL rats to 0.39 ± 0.26 (*p* < 0.001). In Add muscles changes were similar to those in TA muscle. After Cypr the EMG burst amplitude decreased to 0.37 ± 0.17 (*p* < 0.0001) in UL rats and to 0.28 ± 0.25 (*p* < 0.0001) in LL rats. After Volin application, the amplitude was reduced significantly only in LL rats to 0.35 ± 0.15 (*p* < 0.001) (Figure 4C). At the same time, the post hoc Dunn’s multiple comparisons demonstrated that there was no difference in the level of EMG reduction after application of either drug in either group of rats (*p* > 0.05). Moreover, IT application of SB 242 and SB 206 did not induce any significant changes in the amplitude of any muscle (*p* > 0.05) and amplitude of investigated muscle activity during locomotion was significantly higher than that after Cypr and Volin applications (see Figures 4A–C).

Thus, the application of Cypr or Volin reduced the amplitude of EMG activity during locomotion in all muscles. Cypr application evoked a decrease of EMG amplitude in all muscles in UL as well as LL rats. Volin application was less effective than the Cypr and caused a significant decrease of EMG amplitude in Add and TA muscles only in LL rats; while Sol EMG burst activity was reduced in UL rats only. These results suggest direct actions of the 5-HT_2A_ receptor antagonists on motoneurons or pre-motoneurons, with greater effects when the cannula tip was located in closer proximity to the motoneuron pool under investigation. Moreover, these results suggest that EMG amplitude is controlled by receptors other than 5-HT_2A_ receptors since the effect of Cypr was present in UL and LL rats while Volin significantly affected EMG mainly in LL rats. However, the reduction of EMG amplitude was not due to the blockage of 2-HT_2B//2C_ receptors due to lack of any effect of SB 242 or SB 206 IT applications.

### Inter- and Intra-Limb Coordination

**FIGURE 5:**
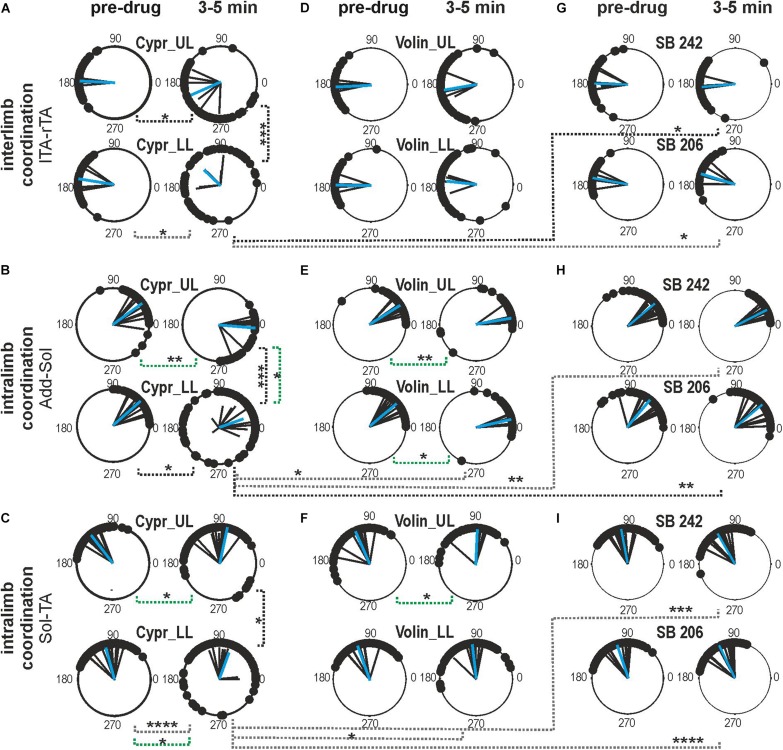
Inter- and intralimb coordination after blockade of various 5-HT receptors by either Cyproheptadine **(A–C)**, Volinanserin **(D–F)**, SB 242084 or SB 206553 **(G–I)** application in the upper lumbar (UL) or low lumbar (LL) spinal cord. **(A,D,G)** Polar plots show interlimb (established between left *vs*. right TA muscles) and **(B,C,E,F,H,I)** intralimb coordination – black vectors are established for each pair of muscles in individual animals; blue vectors are the mean for the group of animals. Particular dots in the polar plots illustrate the phase shift between analyzed EMG bursts of two different muscle onsets: interlimb coordination (lTA *vs*. rTA) or intralimb coordination (Add *vs*. Sol or Sol *vs*. TA). Concentrated (*vs.* dispersed) distribution of single dots on the polar plots confirms strong (*vs.* weak) coupling in the two analyzed muscle EMG bursts. The length of the vector (***r***, ranging from 0 to 1) indicates the strength of coordination between analyzed muscle burst onsets demonstrating the concentration of dots around the mean. The angle of the ***r***-vector illustrates the phase shift between analyzed muscle burst onsets of particular muscle coordination. For more details, see description in the “Materials and Methods” section. Note the well pronounced effect of Cypr in contrast to lack of an effect of Volin in interlimb coordination after application in the low lumbar segments (**A** *vs.* **D**). The strength of adductor *vs.* soleus intralimb coordination **(B,E)** and soleus *vs.* tibialis anterior intralimb coordination **(C,F)** was significantly affected by Cypr in LL rats but not by Volin or by SB 242 and SB 206 drug application (black doted lines). The phase shift was significantly altered only by Cypr in UL and LL rats (green doted lines), but not by Volin or SB 242 and SB 206. Statistical significance for difference of the strength and phase shift of inter- and intralimb coordination was established in the different groups of animals using nonparametric Kruskal-Wallis test followed by Dunn’s multiple comparisons (*p < 0.05; **p < 0.01; ***p < 0.001; ****p < 0.0001). Results from left and right muscles were pooled in all the rats in the various experimental conditions, so the number of samples taken for analysis were: Cypr_UL *n* = 12, Cypr_LL *n* = 10, Volin_UL *n* = 10, Volin LL *n* = 10, SB 242 *n* = 12, SB 206 *n* = 10. Add, adductor muscle; Sol, soleus muscle; lTA/rTA, left/right tibialis anterior muscle; Cypr, Cyproheptadine; Volin, Volinanserin; SB 242, SB 242084; SB 206, SB 206553; UL, upper lumbar segments (L2/L3); LL, low lumbar spinal cord (L5/L6).

Next, we tested the role of 5-HT_2A_, 5-HT_2B/2C_ receptors on inter- and intra-limb coordination. For the Polar Plot analysis, we choose the same episodes of rhythmic locomotor activity selected for step cycle, burst duration and regression line analysis. The Kruskal–Wallis non-parametric analysis indicated that the strength of interlimb (L-R) coordination (*r*-value), was significantly reduced after Cypr applications in LL and UL rats [from 0.96 ± 0.02 to 0.62 ± 0.16; KW(11) = 34.09, *p* = 0.0003; Dunn’s multiple comparisons *p* < 0.02, for both], while there was no significant difference in the strength of L-R coordination after any other drug (Volin, SB 242 or SB 206) in the other experimental groups (Dunn’s multiple comparisons *p* > 0.05). The difference between the results of Cypr and Volin application indicates that 5-HT_2A_ receptors do not contribute to the control of interlimb coordination (Figure 5).

Investigation of the strength of intralimb coordination indicated a significant decrease in the strength of intralimb coordination after Cypr administration in LL rats but not in UL rats. The strength of Add *–* Sol burst coordination was significantly reduced by Cypr application in LL rats [from 0.98 ± 0.01 to 0.67 ± 0.25; KW(11) = 26.74, *p* = 0.005; uncorrected Dunn’s test *p* = 0.01] but not by Volin (*p >* 0.05). The strength of Sol – TA EMG burst activity coordination was also significantly reduced by Cypr application in LL rats only [from 0.97 ± 0.02 to 0.73 ± 0.19; KW(11) = 48.66, *p* < 0.0001; Dunn’s multiple comparisons *p* < 0.0001]. Volinanserin, SB 242 and SB 206 application did not induce any significant decrement of the strength of intralimb coordination (*p* > 0.05). There was a significant difference in the strength of the intralimb coordination when comparing the effects of Cypr and Volin, Cypr and SB 242 as well as Cypr and SB206 in Sol -TA coordination in LL rats [0.73 ± 0.19 *vs.* 0.92 ± 0.05; KW(5) = 33.13, *p* < 0.0001; Dunn’s multiple comparisons *p* < 0.05, *p* < 0.001, *p* < 0.0001, respectively].

The phase shift of the interlimb coordination was not affected by any drug application (Cypr, Volin, SB 242, SB 206) in any experimental groups [KW(11) = 13.67, *p* = 0.25]. While, the phase shift of intralimb coordination was significantly altered by Cypr and Volin but not by SB 242 and SB 206. The Add - Sol coordination was significantly changed [KW(11) = 56.71, *p* < 0.0001] after Cypr in UL rats (33.3 ± 19.79 *vs.* −4.9 ± 24.62; Dunn’s multiple comparisons *p* < 0.01) and after Volin applications in UL (31.95 ± 12.48 *vs.* 9.71 ± 9.63; Dunn’s multiple comparison *p* < 0.01) and LL rats (42.1 ± 16.36 *vs.* 12.82 ± 7.44; Dunn’s multiple comparison *p* < 0.01). The Sol – TA coordination was affected significantly [KW(11) = 43.3, *p* < 0.0001] after Cypr in UL (126.0 ± 8.46 *vs.* 96.25 ± 24.50; Dunn’s multiple comparison *p* < 0.05) and LL rats (105.2 ± 23.34 *vs.* 69.87 ± 37.45; Dunn’s multiple comparison *p* < 0.05) rats while only in UL rats (113.7 ± 26.32 *vs.* 87.45 ± 19.11; Dunn’s multiple comparison *p* < 0.05) after Volin.

The difference between the Cypr results and those obtained with Volin suggest that Cypr alters the strength of inter-limb coordination by acting on some receptors other than the 5-HT_2A_ receptor, such as the 5-HT_2C_ or 5-HT_2B_ receptor (Murray et al., 2011) or 5-HT_7_ receptor (Klein and Teitler, 2011; Leopoldo et al., 2011). In contrast, both drugs induced substantially different changes in the phase shift of intralimb coordination between extensors as well as between flexor and extensor EMG bursts in both the UL and LL rats. These results may suggest that 5-HT_2A_ receptors are present on flexor - extensor coordinating interneurons as well as on motoneurons.

### Reflex Responses to Blockage of 5-HT Receptors

**FIGURE 6:**
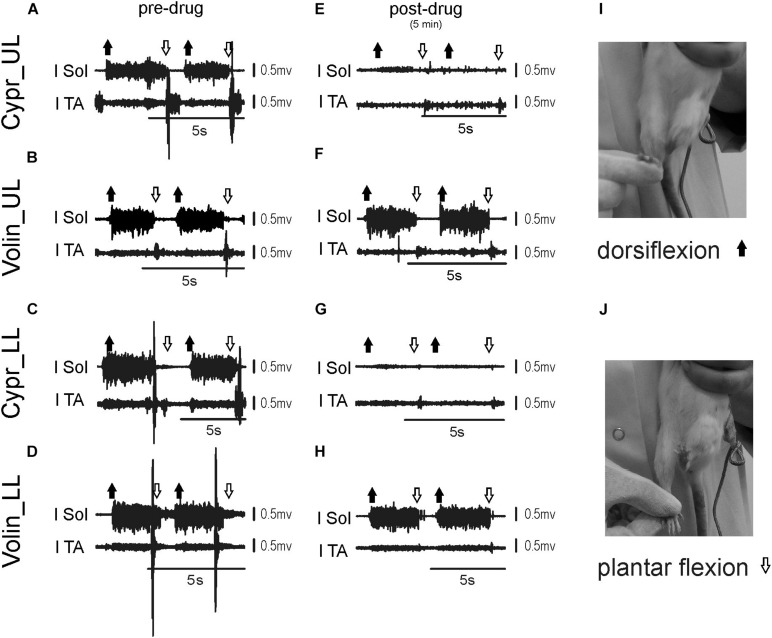
Examples of EMG response of soleus and tibialis anterior muscles to plantar- and dorsiflexion before **(A–D)** and 3–5 min **(E–H)** after intrathecal drug application in the upper (UL) and low (LL) lumbar spinal cord. The examples of two consecutive EMG responses confirm stability of stretch reflexes evoked before and after drug applications. Photos in the right panels **(I,J)** present experimental conditions carried out to induce stretch reflexes in the ankle muscles. Note that on the soleus EMG activity Cyproheptadine but not Volinanserin application had a clear effect. l Sol, left soleus muscle; l TA, left tibialis anterior muscle; Cypr, Cyproheptadine; Volin, Volinanserin; SB 242, SB 242084; SB 206, SB 206553; UL, upper lumbar segments (L2/L3); LL, low lumbar spinal cord (L5/L6).

**FIGURE 7:**
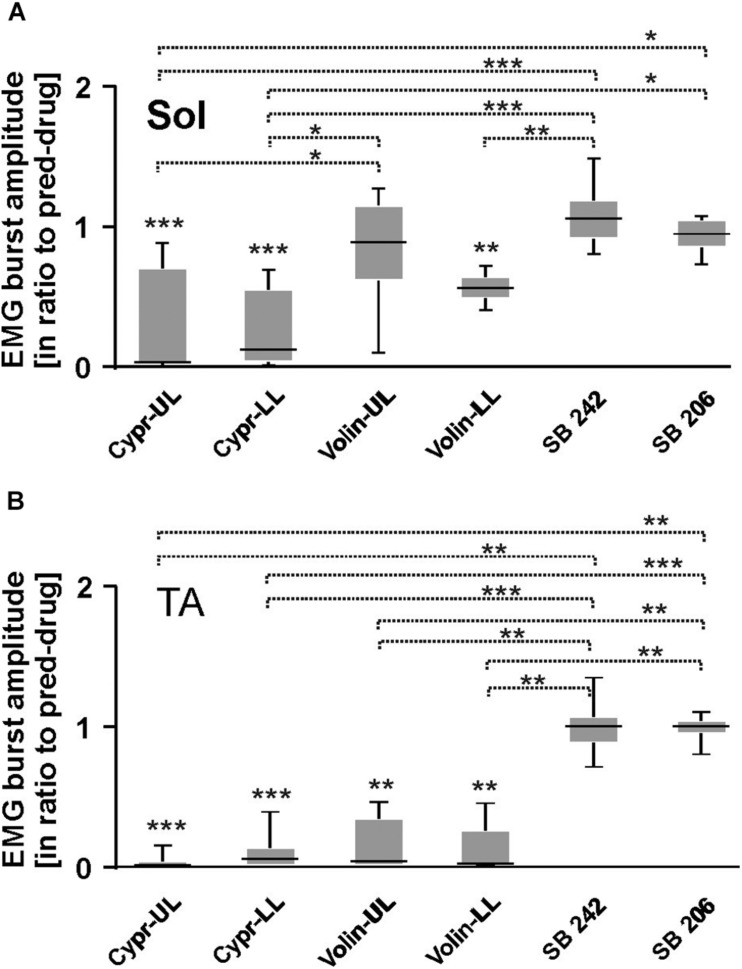
EMG amplitude of soleus **(A)**, tibialis anterior **(B)** muscles before and after 5-HT receptor blockage by either Cyproheptadine, Volinanserin, SB 242084 or SB 206553 application in the upper (UL) or low (LL) lumbar spinal cord during plantar- and dorsiflexion established for Sol and TA muscles of both hindlimbs. Note that in the case of soleus EMG activity Cyproheptadine but not Volinanserin application reduced significantly the reflex response to dorsiflexion in the UL rats **(A)**, while TA EMG response to plantarflexion was reduced by either drug application in both UL and LL rats **(B)**. Results from left and right muscles were pooled in all the rats in the various experimental conditions, so the number of samples taken for analysis were: Cypr UL *n* = 12, Cypr LL *n* = 10, Volin UL *n* = 10, Volin LL *n* = 10, SB 242 *n* = 12, SB 206 *n* = 10. Sol, soleus muscle; TA, tibialis anterior muscle; Cypr, Cyproheptadine; Volin, Volinanserin; SB 242, SB 242084; SB 206, SB 206553; UL, upper lumbar segments (L2/L3); LL, low lumbar spinal cord (L5/L6). The significance of the results was tested using non-parametric Kruskal–Wallis test followed by Dunn’s multiple comparisons (*p < 0.05, **p < 0.01, ***p < 0.001).

Figures 6A–H represents examples of EMG recordings during dorsi- and plantar flexion of the ankle joint, which induces stretch reflexes in the Sol and TA muscles (see the I and J photos in Figure 6 showing a way to induce ankle muscles stretch reflexes). In intact awake animals foot dorsiflexion (Figures 6A–D) evokes a long response with high amplitude in Sol muscle (extensor). Foot plantar flexion abruptly terminates the response of Sol muscle induced by dorsiflexion and induces a short response with high amplitude in the TA muscle (Figures 6A–D). Intrathecal application of Cypr induces a clear reduction of EMG responses to muscle stretch in about 3–5 min after IT drug applications in UL and LL rats (Figures 6E,G). The effect of the Volin application was less pronounced since did not change the amplitude of Sol EMG (Figures 6F,H). IT application of SB 242 or SB 206 did not affected the responses to stretch in either muscle (EMG examples not shown). Administration of either a 5-HT_2A_ receptor neutral antagonist (Volin) or a 5-HT_2A_ receptor inverse agonist (Cypr) caused significant changes of the amplitude of EMG burst activity of the Sol muscles recorded during ankle dorsiflexion [KW(11) = 69.73, *p* < 0.0001; see Figure 7A]. Dunn’s test for multiple comparisons showed that the application of Cypr evoked a decrease of the Sol EMG amplitude both in UL and LL rats to 0.26 ± 0.37 (*p* < 0.001) and to 0.33 ± 0.35 (*p* < 0.01) respectively. Application of Volin induced a decrease of the Sol EMG amplitude only in the LL rats to 0.56 ± 0.1 (*p* < 0.01). Moreover, in the UL rats the Sol EMG burst amplitude was significantly lower after Cypr than after Volin (0.26 ± 0.37 *vs.* 0.83 ± 0.40, *p* < 0.05). Additionally our results demonstrate that application of neither SB 242 nor SB 206 had any effect on the response of Sol muscle to dorsiflexion (*p* > 0.05 for either drug). In TA muscles (Figure 7B) administration of both 5-HT_2A_ receptor blockers also evoked a decrease of the EMG amplitude during plantar flexion at the ankle joint [KW(11) = 86.26, *p* < 0.0001]. Dunn’s test for multiple comparisons revealed that significant changes were present in UL as well as LL rats. After Cypr the TA EMG amplitude decreased in UL rats to 0.03 ± 0.05 (*p* < 0.001) and in LL rats to 0.09 ± 0.12 (*p* < 0.001) while after Volin to 0.17 ± 0.19 in UL rats (*p* < 0.01) and to 0.12 ± 0.18 (*p* < 0.01) in LL rats. Moreover, our results demonstrate that application of neither SB242 nor SB206 affected the response of TA muscle to plantar flexion (*p* > 0.05 for either drug).

All these results on the effects of 5-HT_2B/2C_ receptor blockade confirm the absence of any significant effect of either drug and support the conclusion that 5-HT_2B/2C_ receptors do not make a significant contribution to the control of Central Pattern Generator neurons in the uninjured spinal cord. In Sol muscle both Cypr and Volin decreased EMG amplitude in muscle stretch reflex response (monosynaptic reflex) in LL rats, but in UL rats only Cypr evoked a decrease of Sol EMG amplitude. These results are consistent with the presence of 5-HT_2A_ receptors on motoneurons. Plantar flexion reflex in TA muscle (polysynaptic reflex) was reduced after Cypr and after Volin in LL as well as in UL rats. This result is consistent with the presence of 5-HT_2A_ receptors on interneurons. No effects of Volin in UL rats in Sol motoneuron actions during stretch reflexes confirm our previous hypothesis that the cycle prolongation observed after blockade of 5-HT_2A_ might be due to action on the locomotor CPG.

## Discussion

The current results confirm our previous demonstration that the IT Cypr application produces hindlimb paralysis in adult, intact rats (Majczyński et al., 2005). They provide also additional information regarding the features of the locomotor output that are controlled by 5-HT_2_ receptors. The use of EMG recordings allowed us to monitor 5-HT_2_ actions on the locomotor cycle, burst amplitude, the relationship of burst activity *vs.* cycle durations as well as the inter- (left-right) and intralimb (Sol-TA and Add-Sol) coordination. Effects on motoneuron excitability using stretch reflexes were also investigated. The mechanism of its action seems to be more complex than action on 5-HT_2A_ receptors only. We compared the effects of drugs applied at the UL and LL sites (upper and low lumbar segments) and were able in some cases to separate effects on the rhythm generating (UL) and output elements (LL) of the locomotor system.

### Spinal 5-HT_2A_ Receptor Control of Hindlimb Locomotion

Cypr has effects on multiple receptor types, while Volin, a 5-HT_2A_ neutral antagonist, is more specific for the 5-HT_2A_ receptor. The two drugs had similar effects in most cases, except that Cypr reduced EMG amplitude in the muscles in both UL and LL rats while Volin application reduced EMG amplitude mainly in LL rats. This could be a result of the MN pool location for TA and Add, but this was not the case because Volin did not have the same effect. In contrast to the effects of Cypr and Volin, SB 242 and SB 206 application did not affect EMG amplitude in any muscles. These results suggest direct actions of the 5-HT_2A_ receptor antagonists on motoneurons, with greater effects when the cannula tip was located in closer proximity to the motoneuron pool under investigation. L2/L3 spinal segments contain Add motoneurons and some of those innervating TA muscle, while L4/L5 segments contain the majority of motoneurons innervating Sol muscle and some of those innervating TA muscle (Nicolopoulos-Stournaras and Iles, 1984). Moreover, these results suggest that EMG amplitude is controlled by receptors other than 5-HT_2A_ receptors since the effect of Cypr was present in UL and LL rats while Volin significantly affected EMG mainly in LL rats. We suggest that it might be an effect on pattern formation interneurons with 5-HT_7_ receptors, to which Cypr has affinity as well (Table 1). In addition, our results demonstrate that the 5-HT_2B/2C_ receptors are not involved in control of the EMG amplitude of Sol, Add and TA muscles during locomotion in intact adult rats.

The strength of left-right coordination was affected only by Cypr in LL and UL rats, while Volin was without such effect. Both drugs altered Sol-TA and Add-Sol coordination at in UL rats. In LL rats Cypr disruption of Sol-TA and Add-Sol coordination was pronounced in reduction of the strength of coordination, while Volin had no such effect. This suggests that receptors other than 5-HT_2A_ are affected by Cypr to control strength of inter- and intra-coordination. Our results indicate that the changes in left-right coordination in LL and UL rats after Cypr are not the result of its 5-HT_2B/2C_ receptor component. Considering the affinity of Cypr to 5-HT_7_ receptors and our previous results after SB 269970 (Cabaj et al., 2017), we suggest that changes in interlimb coordination produced by Cypr may be related to the same receptor mechanism. However, further experiments are needed to explore this issue, because Cypr has a very wide range of action, and other mechanisms, for example, dopaminergic or cholinergic receptor interactions, can play a role.

If we assume the LL site is more likely to alter motoneurons and pattern formation neurons (McCrea and Rybak, 2008), we can suggest the interneurons controlling Sol-TA and Add-Sol coordination are in the vicinity of the L5/L6 spinal segments.

The response to stretch induced by dorsiflexion of the ankle joint (monosynaptic reflex) recorded in the EMG of Sol muscle was reduced by Cypr in both UL and LL rats but by Volin only in LL rats. The stretch response of the TA muscle induced by plantar flexion at the ankle joint was reduced by both Cypr and Volin application in UL as well as in LL rats. In contrast to these, administration of 5-HT_2B/2C_ receptor ligands did not affect the EMG amplitude of neither the Sol nor TA muscles. These results affecting monosynaptic responses in stretch reflex are consistent with the presence of 5-HT_2A_ receptors on motoneurons at the LL level. The Cypr and Volin effects in UL sites confirm that 5-HT_2A_ receptors are also present on interneurons. However, the efficacy of Cypr in the UL level might be also due to an action mediated by its affinity for 5-HT_7_ receptors (Leopoldo et al., 2011), because some of the locomotor interneurons with 5-HT_7_ receptors may project directly to motoneurons. From the other hand, Cypr could be exerting its effect due to its affinity to 5-HT_2B/2C_ receptors. However, it is not the case because our investigations demonstrated clearly that in adult intact rats, neither 5-HT_2B_ nor 5-HT_2C_ receptors appear to play a role in determining motoneuron excitability. Further experiments are needed to explain this issue.

### Lack of an Effect of Blockage of 5-HT_2B_ or 5-HT_2C_ Receptors

We used the neutral antagonist of the 5-HT_2C_ receptor (SB 242084) and the inverse agonist of 5-HT_2B/2C_ (SB 206993) and showed that these compounds did not produce any significant alterations in the locomotor and reflex activity. In our preliminary experiments, we used the doses of both blockers based on those used in adult rats with staggered hemisection by Fouad et al. (2010). When those doses (3 mM; 30 μl) in our experiments did not show any locomotor alteration we doubled the doses (6 mM; 30 μl) but this did not induce any significant effects either. Thus, our results demonstrated that neutral antagonist, as well as inverse agonist, did not affect the locomotor activity in intact rats.

The apparent importance of constitutively active 5-HT receptors in the recovery of locomotor activity and motoneuron excitability after spinal cord injury (Fouad et al., 2010; Murray et al., 2010) is considered one of the most significant contributions to the understanding of the fact that locomotion reappears after spinal cord injury, despite removal of the endogenous ligand, 5-HT, as a result of SCI. Our results provide a necessary test of the possibility that 5-HT_2C_ receptor activity contributes to locomotion in the normal animal. Measures used in the past included the BBB score (Basso et al., 1996), which ignores left-right coordination completely and has no implicit measure of flexor-extensor coordination in the hindlimbs. These are aspects of plantar stepping now known to be controlled by 5-HT (Sławińska et al., 2014b) that we tested in our current experiments. In particular, EMG amplitude and step cycle duration, as well as coordination, are known to be controlled by 5-HT. Our data shows that activity in 5-HT_2B/2C_ receptors does not contribute to the control of any aspect of locomotion that we recorded.

Fouad et al. (2010) and Murray et al. (2010) provide data testing the effects of 5-HT_2C_ antagonists in locomotion, using a staggered hemisection model and IT injection into the cauda equina level. It is possible that drugs applied at this site acted on input from the sacral level (Lev-Tov et al., 2000), which is known to induce locomotion. The drugs used in this fashion may not have penetrated to the level of the spinal cord controlling locomotion, leaving negative data difficult to interpret. This is the case where no effect of the neutral antagonist SB 242084 is used as a definitive test for constitutively active 5-HT_2C_ receptor involvement in locomotor recovery after SCI (Fouad et al., 2010; Murray et al., 2010). If the relevant cells in the locomotor system can be missed using a sacral injection, then results obtained using a sacral injection to target the locomotor CPG must be interpreted with caution. We have avoided this problem using the lidocaine test of effective drug delivery in any case where a negative result was obtained.

It is not immediately obvious why receptors not normally employed in the control of locomotion should suddenly become essential after injury. According to Murray et al. (2010), there are few 5-HT neurons in the brainstem that provide the extensive innervation of spinal motoneurons and interneurons, so these neurons are vulnerable to injury or disease that decreases activity in the few 5-HT neurons. They suggest that constitutive 5-HT_2_ receptor activity “…provides a safeguard against such loss of 5-HT innervation of the spinal cord and probably even contributes to basal receptor activity in normal rats.” Our results do not provide confirmation of any contribution of constitutive activity in 5-HT_2_ receptors to basal receptor activity in normal rats.

It is well known that 5-HT_2C_ receptor plasticity may account for serotonin supersensitivity of V2a interneurons after spinal cord injury (Husch et al., 2012). This is of interest because V2a interneurons are likely components of the CPG for locomotion. These neurons are recruited with increases in locomotor speed (Zhong et al., 2011), and the deletion of their effects disrupts locomotion at higher speeds (Crone et al., 2008). Our data shows that the normal situation differs from the acute and chronic spinal condition with respect to 5HT_2C_ receptor function.

### Role of 5-HT Receptor Types Regarding Their Rostro-Caudal Location

5-HT application to the isolated spinal cord induces locomotor-like discharge of lumbar ventral roots (Kudo and Yamada, 1987a, b; Cazalets et al., 1990, 1992; Sqalli-Houssaini et al., 1993; Cowley and Schmidt, 1994; Kjaerulff and Kiehn, 1996; Beato et al., 1997), with a rostro-caudal decrease in excitability of the locomotor system. There is evidence showing that the CPG network is localized in upper lumbar segments, while the pattern formation interneurons are present closer to the motoneuron pools innervating limb muscles. There are further data suggesting a regional differentiation of certain receptors in different rostro-caudal regions (Jordan and Schmidt, 2002; Liu and Jordan, 2005; Liu et al., 2009) that are in line with the differential effects of 5-HT on supra-lumbar *vs.* lumbar regions. Our use of UL and LL cannula tip placements was guided by these findings, and our results provide new insights into the organization of the spinal locomotor network. In particular, our results show that the effects of drugs applied at the UL and LL sites were able to separate effects on the rhythm generating (UL) *vs.* motoneurons and pattern formation (LL) elements of the locomotor system. The regional distribution of effective receptors, along with their association with identified components of the locomotor system, can be revealed by localized application of antagonists and inverse agonists. This issue should be investigated further.

## Data Availability Statement

The datasets generated for this study are available on request to the corresponding author.

## Ethics Statement

The animal study was reviewed and approved by The First Ethics Committee for Animal Experimentation in Poland.

## Author Contributions

HM, US, and LJ conceived and designed the experiments. HM, AC, and US performed the experiments conducted in the Laboratory of Neuromuscular Plasticity in the Nencki Institute of Experimental Biology of PAS and performed the EMG data analysis. HM, US, and LJ participated in the discussion of results and wrote the manuscript. All authors approved the final version of the manuscript, all persons designated as authors qualify for authorship, and all those who qualify for authorship are listed.

## Conflict of Interest

The authors declare that the research was conducted in the absence of any commercial or financial relationships that could be construed as a potential conflict of interest.

## Footnotes

1 http://www.scrc.umanitoba.ca/doc/

2 www.graphpad.com

## Supplementary Material

FIGURE S1 Examples of EMG activity in muscles of adult freely moving rats before and after either neutral antagonist – SB 242084 **(A–C)** or inverse agonist – SB 206553 **(B–D)** application in the lumbar spinal cord. **(A,B)** Illustrate the EMG activity recorded during locomotion along the horizontal runway in adult freely moving rats in the control pre-drug situation. **(C,D)** Illustrate examples of EMG activity recorded 3–5 min after the drug administration. Note the similar pattern and amplitude of EMG activity before and after drug applications. These examples show that the rats used 12–14 steps to pass the 2 m long runway and such recording periods were taken for further quantifications of locomotor performance. lAdd/rAdd, left/right adductor muscle; lSol/rSol, left/right soleus muscle; lTA/rTA, left/right tibialis anterior muscle; lTri/rTri, left/right triceps brachii muscle.

FIGURE S2 The relationship between Sol EMG burst duration *vs.* step cycle duration before and after Cyproheptadine **(A),** Volinanserin **(B)** and SB 242084 or SB 206553 **(C)** applications in the upper lumbar (UL) or low lumbar (LL) spinal cord established using regression line analysis. Each regression line illustrated by a given color was established based on consecutive EMG cycles and associated EMG burst duration and illustrated in the drawing as a single dot with particular XY coordinates. Note that in the control situation and in cases of lack of drug-induced locomotor alterations the dots are distributed in a concentrated manner very close to the established regression line. While in a case of locomotor deteriorations the dots are dispersed in some distance from the regression lines confirming the lack of normal relationships between EMG burst and cycle duration. In the right panel **(D)** the bars illustrate the mean (±SD) of regression line slopes established for hindlimb muscles in different experimental conditions. The significance of differences in the slopes in various groups of animals before and after various treatment was tested using nonparametric Kruskal–Wallis test followed by Dunn’s test for multiple comparisons (**p <* 0.05, ***p <* 0.01, ****p <* 0.001, *****p <* 0.0001). Results from left and right muscles were pooled in all the rats in the various experimental conditions, so the number of samples taken for analysis were: Cypr UL *n* = 12, Cypr LL *n* = 10, Volin UL *n* = 10, Volin LL *n* = 10, SB 242 *n* = 12, SB 206 *n* = 10. lSol/rSol, left/right soleus muscle.

FIGURE S3 The relationship between TA EMG burst duration *vs.* step cycle duration before and after Cyproheptadine **(A),** Volinanserin **(B)** and SB 242084 or SB 206553 **(C)** applications in the upper lumbar (UL) or low lumbar (LL) spinal cord established using regression line analysis. In the right panel **(D)** the bars illustrate the mean (±SD) of regression line slopes established for hindlimb muscles in different experimental conditions. The significance of differences in the slopes in various groups of animals before and after various treatment was tested using nonparametric Kruskal–Wallis test followed by Dunn’s test for multiple comparisons (**p <* 0.05, ***p <* 0.01, ****p <* 0.001, *****p <* 0.0001). Results from left and right muscles were pooled in all the rats in the various experimental conditions, so the number of samples taken for analysis were: Cypr UL *n* = 12, Cypr LL *n* = 10, Volin UL *n* = 10, Volin LL *n* = 10, SB 242 *n* = 12, SB 206 *n* = 10. lTA/rTA, left/right tibialis anterior muscle.

FIGURE S4 The relationship between Add EMG burst duration *vs.* step cycle duration before and after Cyproheptadine **(A),** Volinanserin **(B)** and SB 242084 or SB 206553 **(C)** applications in the upper lumbar (UL) or low lumbar (LL) spinal cord established using regression line analysis. In the right panel **(D)** the bars illustrate the mean (±SD) of regression line slopes established for hindlimb muscles in different experimental conditions. The significance of differences in the slopes in various groups of animals before and after various treatment was tested using nonparametric Kruskal–Wallis test followed by Dunn’s test for multiple comparisons (**p <* 0.05, ***p <* 0.01, ****p <* 0.001, *****p <* 0.0001). Results from left and right muscles were pooled in all the rats in the various experimental conditions, so the number of samples taken for analysis were: Cypr UL *n* = 12, Cypr LL *n* = 10, Volin UL *n* = 10, Volin LL *n* = 10, SB 242 *n* = 12, SB 206 *n* = 10. lAdd/rAdd, left/right adductor muscle.

VIDEO S1 Example of rat locomotor performance on a runway in pre-drug conditions.

VIDEO S2 Example of hindlimb movement deteriorations in a rat from UL group after Cyproheptadine IT application.

VIDEO S3 Example of hindlimb movement deteriorations in a rat from LL group after Cyproheptadine IT application.

VIDEO S4 Example of hindlimb movement deteriorations in a rat from UL group after Volinanserin IT application.

VIDEO S5 Example of hindlimb movement deteriorations in a rat from LL group after Volinanserin IT application.

VIDEO S6 Example of locomotor performance on a runway of a rat after SB 242084 IT application.

VIDEO S7 Example of locomotor performance on a runway of a rat after SB 206553 IT application.

## References

[B1] Anglister L. Cherniak M. Lev-Tov A. (2017). Ascending pathways that mediate cholinergic modulation of lumbar motor activity. *J. Neurochem.* 142 (Suppl. 2), 82–89. 10.1111/jnc.14065 28791705

[B2] Anglister L. Etlin A. Finkel E. Durrant A. R. Lev-Tov A. (2008). Cholinesterases in development and disease. *Chem. Biol. Interact.* 175 92–100. 10.1016/j.cbi.2008.04.046 18571632

[B3] Antri M. Barthe J. Y. Mouffle C. Orsal D. (2005). Long-lasting recovery of locomotor function in chronic spinal rat following chronic combined pharmacological stimulation of serotonergic receptors with 8-OHDPAT and quipazine. *Neurosci. Lett.* 384 162–167. 10.1016/j.neulet.2005.04.062 15905027

[B4] Antri M. Mouffle C. Orsal D. Barthe J. Y. (2003). 5-HT1A receptors are involved in short- and long-term processes responsible for 5-HT-induced locomotor function recovery in chronic spinal rat. *Eur. J. Neurosci.* 18 1963–1972. 10.1046/j.1460-9568.2003.02916.x 14622228

[B5] Antri M. Orsal D. Barthe J. Y. (2002). Locomotor recovery in the chronic spinal rat: effects of long-term treatment with a 5-HT2 agonist. *Eur. J. Neurosci.* 16 467–476. 10.1046/j.1460-9568.2002.02088.x 12193190

[B6] Barbeau H. Rossignol S. (1990). The effects of serotonergic drugs on the locomotor pattern and on cutaneous reflexes of the adult chronic spinal cat. *Brain Res. Bull.* 514 55–67. 10.1016/0006-8993(90)90435-e 2357531

[B7] Barbeau H. Rossignol S. (1991). Initiation and modulation of the locomotor pattern in the adult chronic spinal cat by noradrenergic, serotonergic and dopaminergic drugs. *Brain Res. Bull.* 546 250–260. 10.1016/0006-8993(91)91489-n 2070262

[B8] Basso D. M. Beattie M. S. Bresnahan J. C. (1996). Graded histological and locomotor outcomes after spinal cord contusion using the NYU weight-drop device versus transection. *Exp. Neurol.* 139 244–256. 10.1006/exnr.1996.0098 8654527

[B9] Batschelet E. (1981). *Circular Statustucs in Biology.* New York, NY: Academic Press.

[B10] Beato M. Bracci E. Nistri A. (1997). Contribution of NMDA and non-NMDA glutamate receptors to locomotor pattern generation in the neonatal rat spinal cord. *Proc. Biol. Sci.* 264 877–884. 10.1098/rspb.1997.0122 9225479 PMC1688428

[B11] Cabaj A. M. Majczyński H. Couto E. Gardiner P. F. Stecina K. Sławińska U. (2017). Serotonin controls initiation of locomotion and afferent modulation of coordination via 5-HT7 receptors in adult rats. *J. Physiol.* 595 301–320. 10.1113/JP272271 27393215 PMC5199736

[B12] Cazalets J. R. Borde M. Clarac F. (1995). Localization and organization of the central pattern generator for hindlimb locomotion in newborn rat. *J. Neurosci.* 15 4943–4951. 10.1523/jneurosci.15-07-04943.1995 7623124 PMC6577873

[B13] Cazalets J. R. Grillner P. Menard I. Cremieux J. Clarac F. (1990). Two types of motor rhythm induced by NMDA and amines in an in vitro spinal cord preparation of neonatal rat. *Neurosci. Lett.* 111 116–121. 10.1016/0304-3940(90)90354-c 2186309

[B14] Cazalets J. R. Sqalli-Houssaini Y. Clarac F. (1992). Activation of the central pattern generators for locomotion by serotonin and excitatory amino acids in neonatal rat. *J. Physiol.* 455 187–204. 10.1113/jphysiol.1992.sp019296 1362441 PMC1175639

[B15] Courtine G. Gerasimenko Y. van den Brand R. Yew A. Musienko P. Zhong H. (2009). Transformation of nonfunctional spinal circuits into functional states after the loss of brain input. *Nat. Neurosci.* 12 1333–1342. 10.1038/nn.2401 19767747 PMC2828944

[B16] Cowley K. C. MacNeil B. J. Chopek J. W. Sutherland S. Schmidt B. J. (2015). Neurochemical excitation of thoracic propriospinal neurons improves hindlimb stepping in adult rats with spinal cord lesions. *Exp. Neurol.* 264 174–187. 10.1016/j.expneurol.2014.12.006 25527257

[B17] Cowley K. C. Schmidt B. J. (1994). A comparison of motor patterns induced by N-methyl-D-aspartate, acetylcholine and serotonin in the in vitro neonatal rat spinal cord. *Neurosci. Lett.* 171 147–150. 10.1016/0304-3940(94)90626-2 8084477

[B18] Cowley K. C. Zaporozhets E. Maclean J. N. Schmidt B. J. (2005). Is NMDA receptor activation essential for the production of locomotor-like activity in the neonatal rat spinal cord? *J. Neurophysiol.* 94 3805–3814. 10.1152/jn.00016.2005 16120672

[B19] Crone S. A. Quinlan K. A. Zagoraiou L. Droho S. Restrepo C. E. Lundfald L. (2008). Genetic ablation of V2a ipsilateral interneurons disrupts left-right locomotor coordination in mammalian spinal cord. *Neuron* 60 70–83. 10.1016/j.neuron.2008.08.009 18940589

[B20] Finkel E. Etlin A. Cherniak M. Mor Y. Lev-Tov A. Anglister L. (2014). Neuroanatomical basis for cholinergic modulation of locomotor networks by sacral relay neurons with ascending lumbar projections. *J. Comp. Neurol.* 522 3437–3455. 10.1002/cne.23613 24752570

[B21] Fouad K. Rank M. M. Vavrek R. Murray K. C. Sanelli L. Bennett D. J. (2010). Locomotion after spinal cord injury depends on constitutive activity in serotonin receptors. *J. Neurophysiol.* 104 2975–2984. 10.1152/jn.00499.2010 20861436 PMC3007654

[B22] Frigon A. Gossard J. P. (2009). Asymmetric control of cycle period by the spinal locomotor rhythm generator in the adult cat. *J. Physiol.* 587 4617–4628. 10.1113/jphysiol.2009.176669 19675066 PMC2768017

[B23] Gerasimenko Y. P. Ichiyama R. M. Lavrov I. A. Courtine G. Cai L. Zhong H. (2007). Epidural spinal cord stimulation plus quipazine administration enable stepping in complete spinal adult rats. *J. Neurophysiol.* 98 2525–2536. 10.1152/jn.00836.2007 17855582

[B24] Ghosh M. Pearse D. D. (2014). The role of the serotonergic system in locomotor recovery after spinal cord injury. *Front. Neural Circ.* 8:151. 10.3389/fncir.2014.00151 25709569 PMC4321350

[B25] Halbertsma J. M. (1983). The stride cycle of the cat: the modelling of locomotion by computerized analysis of automatic recordings. *Acta Physiol. Scand. Suppl.* 521 1–75. 6582764

[B26] Hayashi Y. Jacob-Vadakot S. Dugan E. A. McBride S. Olexa R. Simansky K. (2010). 5-HT precursor loading, but not 5-HT receptor agonists, increases motor function after spinal cord contusion in adult rats. *Exp. Neurol.* 221 68–78. 10.1016/j.expneurol.2009.10.003 19840787 PMC2812640

[B27] Hnik P. Vejsada R. Kasicki S. (1981). Reflex and locomotor changes following unilateral deafferentation of rat hind limb assessed by chronic electromyography. *Neuroscience* 6 195–203. 10.1016/0306-4522(81)90055-5 7219712

[B28] Hnik P. Vejsada R. Kasicki S. (1982). EMG changes in rat hind limb muscles following bilateral deafferentation. *Pflugers Arch. Eur. J. Physiol.* 395 182–185. 10.1007/bf00584806 6891455

[B29] Hounsgaard J. Hultborn H. Jespersen B. Kiehn O. (1988). Bistability of alpha-motoneurones in the decerebrate cat and in the acute spinal cat after intravenous 5-hydroxytryptophan. *J. Physiol.* 405 345–367. 10.1113/jphysiol.1988.sp017336 3267153 PMC1190979

[B30] Husch A. Van Patten G. N. Hong D. N. Scaperotti M. M. Cramer N. Harris-Warrick R. M. (2012). Spinal cord injury induces serotonin supersensitivity without increasing intrinsic excitability of mouse V2a interneurons. *J. Neurosci.* 32 13145–13154. 10.1523/jneurosci.2995-12.2012 22993431 PMC3506248

[B31] Jordan L. M. McVagh J. R. Noga B. R. Cabaj A. M. Majczyński H. Sławińska U. (2014). Cholinergic mechanisms in spinal locomotion-potential target for rehabilitation approaches. *Front. Neural Circ.* 8:132. 10.3389/fncir.2014.00132 25414645 PMC4222238

[B32] Jordan L. M. Schmidt B. J. (2002). Propriospinal neurons involved in the control of locomotion: potential targets for repair strategies? *Prog. Brain Res.* 137 125–139. 10.1016/s0079-6123(02)37012-2 12440364

[B33] Kiehn O. Eken T. (1998). Functional role of plateau potentials in vertebrate motor neurons. *Curr. Opin. Neurobiol.* 8 746–752. 10.1016/s0959-4388(98)80117-7 9914232

[B34] Kim D. Murray M. Simansky K. J. (2001). The serotonergic 5-HT(2C) agonist m-chlorophenylpiperazine increases weight-supported locomotion without development of tolerance in rats with spinal transections. *Exp. Neurol.* 169 496–500. 10.1006/exnr.2001.7660 11358463

[B35] Kjaerulff O. Kiehn O. (1996). Distribution of networks generating and coordinating locomotor activity in the neonatal rat spinal cord in vitro: a lesion study. *J. Neurosci.* 16 5777–5794. 10.1523/jneurosci.16-18-05777.1996 8795632 PMC6578971

[B36] Klein M. T. Teitler M. (2011). Antagonist interaction with the human 5-HT(7) receptor mediates the rapid and potent inhibition of non-G-protein-stimulated adenylate cyclase activity: a novel GPCR effect. *Br. J. Pharmacol.* 162 1843–1854. 10.1111/j.1476-5381.2010.01194.x 21198551 PMC3081126

[B37] Kudo N. Yamada T. (1987a). Morphological and physiological studies of development of the monosynaptic reflex pathway in the rat lumbar spinal cord. *J. Physiol.* 389 441–459. 10.1113/jphysiol.1987.sp016665 2824763 PMC1192089

[B38] Kudo N. Yamada T. (1987b). N-methyl-D,L-aspartate-induced locomotor activity in a spinal cord-hindlimb muscles preparation of the newborn rat studied in vitro. *Neurosci. Lett.* 75 43–48. 10.1016/0304-3940(87)90072-3 3554010

[B39] Landry E. S. Lapointe N. P. Rouillard C. Levesque D. Hedlund P. B. Guertin P. A. (2006). Contribution of spinal 5-HT1A and 5-HT7 receptors to locomotor-like movement induced by 8-OH-DPAT in spinal cord-transected mice. *Eur. J. Neurosci.* 24 535–546. 10.1111/j.1460-9568.2006.04917.x 16836640

[B40] Langlet C. Leblond H. Rossignol S. (2005). Mid-lumbar segments are needed for the expression of locomotion in chronic spinal cats. *J. Neurophysiol.* 93 2474–2488. 10.1152/jn.00909.2004 15647400

[B41] Leopoldo M. Lacivita E. Berardi F. Perrone R. Hedlund P. B. (2011). Serotonin 5-HT7 receptor agents: structure-activity relationships and potential therapeutic applications in central nervous system disorders. *Pharmacol. Ther.* 129 120–148. 10.1016/j.pharmthera.2010.08.013 20923682 PMC3031120

[B42] Lev-Tov A. Delvolve I. Kremer E. (2000). Sacrocaudal afferents induce rhythmic efferent bursting in isolated spinal cords of neonatal rats. *J. Neurophysiol.* 83 888–894. 10.1152/jn.2000.83.2.888 10669502

[B43] Liu J. Akay T. Hedlund P. B. Pearson K. G. Jordan L. M. (2009). Spinal 5-HT7 receptors are critical for alternating activity during locomotion: in vitro neonatal and in vivo adult studies using 5-HT7 receptor knockout mice. *J. Neurophysiol.* 102 337–348. 10.1152/jn.91239.2008 19458153

[B44] Liu J. Jordan L. M. (2005). Stimulation of the parapyramidal region of the neonatal rat brain stem produces locomotor-like activity involving spinal 5-HT7 and 5-HT2A receptors. *J. Neurophysiol.* 94 1392–1404. 10.1152/jn.00136.2005 15872068

[B45] Majczyński H. Cabaj A. Górska T. (2005). Intrathecal application of cyproheptadine impairs locomotion in intact rats. *Neurosci. Lett.* 381 16–20. 10.1016/j.neulet.2005.01.075 15882782

[B46] Majczyński H. Cabaj A. Sławińska U. Górska T. (2006). Intrathecal administration of yohimbine impairs locomotion in intact rats. *Behav. Brain Res.* 175 315–322. 10.1016/j.bbr.2006.08.040 17010450

[B47] Majczyński H. Maleszak K. Górska T. Sławińska U. (2007). Comparison of two methods for quantitative assessment of unrestrained locomotion in the rat. *J. Neurosci. Meth.* 163 197–207. 10.1016/j.jneumeth.2007.02.023 17418901

[B48] McCrea D. A. Rybak I. A. (2008). Organization of mammalian locomotor rhythm and pattern generation. *Brain Res. Rev.* 57 134–146. 10.1016/j.brainresrev.2007.08.006 17936363 PMC2214837

[B49] Murray K. C. Nakae A. Stephens M. J. Rank M. D’Amico J. Harvey P. J. (2010). Recovery of motoneuron and locomotor function after spinal cord injury depends on constitutive activity in 5-HT2C receptors. *Nat. Med.* 16 694–700. 10.1038/nm.2160 20512126 PMC3107820

[B50] Murray K. C. Stephens M. J. Ballou E. W. Heckman C. J. Bennett D. J. (2011). Motoneuron excitability and muscle spasms are regulated by 5-HT2B and 5-HT2C receptor activity. *J. Neurophysiol.* 105 731–748. 10.1152/jn.00774.2010 20980537 PMC3059173

[B51] Musienko P. van den Brand R. Marzendorfer O. Roy R. R. Gerasimenko Y. Edgerton V. R. (2011). Controlling specific locomotor behaviors through multidimensional monoaminergic modulation of spinal circuitries. *J. Neurosci.* 31 9264–9278. 10.1523/jneurosci.5796-10.2011 21697376 PMC3422212

[B52] Nicolopoulos-Stournaras S. Iles J. F. (1983). Motor neuron columns in the lumbar spinal cord of the rat. *J. Comp. Neurol.* 217 75–85. 10.1002/cne.902170107 6875053

[B53] Nicolopoulos-Stournaras S. Iles J. F. (1984). Hindlimb muscle activity during locomotion in the rat (*Rattus norvegicus*) (Rodentia: Murydae). *J. Zool.* 203 427–440. 10.1111/j.1469-7998.1984.tb02342.x

[B54] Schmidt B. J. Jordan L. M. (2000). The role of serotonin in reflex modulation and locomotor rhythm production in the mammalian spinal cord. *Brain Res. Bull.* 53 689–710. 10.1016/s0361-9230(00)00402-0 11165804

[B55] Sławińska U. Jordan L. M. Cabaj A. M. Burger B. Fabczak H. Majczyński H. (2012a). “Locomotion of intact adult rats is controlled by 5-HT2A and 5-HT7 but not 5-HT2C receptors,” in *Program No. 85.09. Neuroscience Meeting Planner* (New Orleans, LA: Society for Neuroscience, 2012).

[B56] Sławińska U. Kasicki S. (2002). Altered electromyographic activity pattern of rat soleus muscle transposed into the bed of antagonist muscle. *J. Neurosci.* 22 5808–5812. 10.1523/jneurosci.22-14-05808.2002 12122041 PMC6757917

[B57] Sławińska U. Majczyński H. Dai Y. Jordan L. M. (2012b). The upright posture improves plantar stepping and alters responses to serotonergic drugs in spinal rats. *J. Physiol.* 590 1721–1736. 10.1113/jphysiol.2011.224931 22351637 PMC3413485

[B58] Sławińska U. Majczyński H. Djavadian R. (2000). Recovery of hindlimb motor functions after spinal cord transection is enhanced by grafts of the embryonic raphe nuclei. *Exp. Brain Res.* 132 27–38. 10.1007/s002219900323 10836633

[B59] Sławińska U. Miazga K. Cabaj A. M. Leszczyńska A. N. Majczyński H. Nagy J. I. (2013). Grafting of fetal brainstem 5-HT neurons into the sublesional spinal cord of paraplegic rats restores coordinated hindlimb locomotion. *Exp. Neurol.* 247 572–581. 10.1016/j.expneurol.2013.02.008 23481546

[B60] Sławińska U. Miazga K. Jordan L. M. (2014a). 5-HT(2) and 5-HT(7) receptor agonists facilitate plantar stepping in chronic spinal rats through actions on different populations of spinal neurons. *Front. Neural Circ.* 8:95. 10.3389/fncir.2014.00095 25191231 PMC4137449

[B61] Sławińska U. Miazga K. Jordan L. M. (2014b). The role of serotonin in the control of locomotor movements and strategies for restoring locomotion after spinal cord injury. *Acta Neurobiol. Exp.* 74 172–187. 10.55782/ane-2014-1983 24993627

[B62] Sławińska U. Navarrete R. Kasicki S. Vrbová G. (1995). Motor activity patterns in rat soleus muscle after neonatal partial denervation. *Neurom Disord.* 5 179–186. 10.1016/0960-8966(94)00053-c 7633182

[B63] Sqalli-Houssaini Y. Cazalets J. R. Clarac F. (1993). Oscillatory properties of the central pattern generator for locomotion in neonatal rats. *J. Neurophysiol.* 70 803–813. 10.1152/jn.1993.70.2.803 8410173

[B64] Tysseling V. M. Klein D. A. Imhoff-Manuel R. Manuel M. Heckman C. J. Tresch M. C. (2017). Constitutive activity of 5-HT2C receptors is present after incomplete spinal cord injury but is not modified after chronic SSRI or baclofen treatment. *J. Neurophysiol.* 118 2944–2952. 10.1152/jn.00190.2017 28877964 PMC5686237

[B65] Ung R. V. Landry E. S. Rouleau P. Lapointe N. P. Rouillard C. Guertin P. A. (2008). Role of spinal 5-HT2 receptor subtypes in quipazine-induced hindlimb movements after a low-thoracic spinal cord transection. *Eur. J. Neurosci.* 28 2231–2242. 10.1111/j.1460-9568.2008.06508.x 19019202

[B66] van den Brand R. Heutschi J. Barraud Q. DiGiovanna J. Bartholdi K. Huerlimann M. (2012). Restoring voluntary control of locomotion after paralyzing spinal cord injury. *Science* 336 1182–1185. 10.1126/science.1217416 22654062

[B67] Vejsada R. Hnik P. Navarrete R. Palecek J. Soukup T. Borecka U. (1991). Motor functions in rat hindlimb muscles following neonatal sciatic nerve crush. *Neuroscience* 40 267–275. 10.1016/0306-4522(91)90189-u 1828867

[B68] Zar J. H. (2010). “Circular distribution,” in *Biostatistical Analysis*, 5th Edn, eds McElroy W. D. Swanson C. P. (Upper Saddle River: Prentice Hall), 605–668.

[B69] Zhong G. Sharma K. Harris-Warrick R. M. (2011). Frequency-dependent recruitment of V2a interneurons during fictive locomotion in the mouse spinal cord. *Nat. Commun.* 2:274. 10.1038/ncomms1276 PMC3597081 21505430

