# Supplementary figures and images for: Contribution of 5-HT2 Receptors to the Control of the Spinal Locomotor System in Intact Rats
Source: Front Neural Circuits. 2020 Apr 24;14:14. doi: 10.3389/fncir.2020.00014 (PMC7212388; doi:10.3389/fncir.2020.00014)

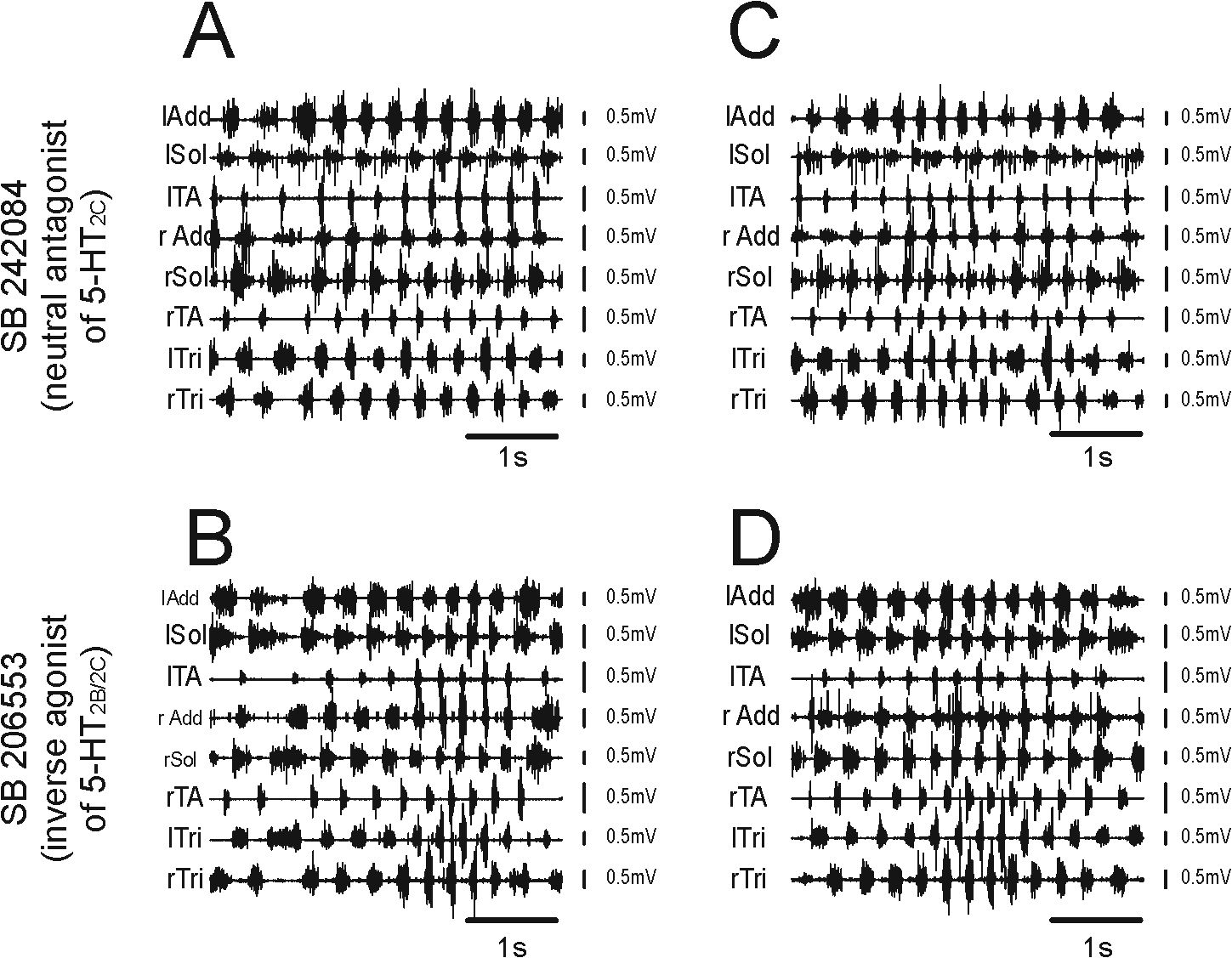

Supplement: FIGURE S1 — Examples of EMG activity in muscles of adult freely moving rats before and after either neutral antagonist – SB 242084 (A–C) or inverse agonist – SB 206553 (B–D) application in the lumbar spinal cord. (A,B) Illustrate the EMG activity recorded during locomotion along the horizontal runway in adult freely moving rats in the control pre-drug situation. (C,D) Illustrate examples of EMG activity recorded 3–5 min after the drug administration. Note the similar pattern and amplitude of EMG activity before and after drug applications. These examples show that the rats used 12–14 steps to pass the 2 m long runway and such recording periods were taken for further quantifications of locomotor performance. lAdd/rAdd, left/right adductor muscle; lSol/rSol, left/right soleus muscle; lTA/rTA, left/right tibialis anterior muscle; lTri/rTri, left/right triceps brachii muscle. [file Image_1.JPEG]

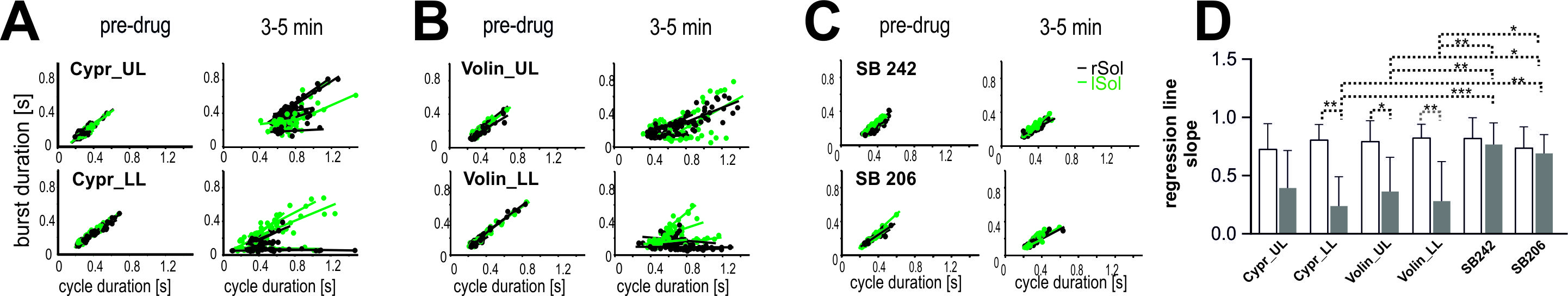

Supplement: FIGURE S2 — The relationship between Sol EMG burst duration vs. step cycle duration before and after Cyproheptadine (A), Volinanserin (B) and SB 242084 or SB 206553 (C) applications in the upper lumbar (UL) or low lumbar (LL) spinal cord established using regression line analysis. Each regression line illustrated by a given color was established based on consecutive EMG cycles and associated EMG burst duration and illustrated in the drawing as a single dot with particular XY coordinates. Note that in the control situation and in cases of lack of drug-induced locomotor alterations the dots are distributed in a concentrated manner very close to the established regression line. While in a case of locomotor deteriorations the dots are dispersed in some distance from the regression lines confirming the lack of normal relationships between EMG burst and cycle duration. In the right panel (D) the bars illustrate the mean (±SD) of regression line slopes established for hindlimb muscles in different experimental conditions. The significance of differences in the slopes in various groups of animals before and after various treatment was tested using nonparametric Kruskal–Wallis test followed by Dunn’s test for multiple comparisons (*p < 0.05, **p < 0.01, ***p < 0.001, ****p < 0.0001). Results from left and right muscles were pooled in all the rats in the various experimental conditions, so the number of samples taken for analysis were: Cypr UL n = 12, Cypr LL n = 10, Volin UL n = 10, Volin LL n = 10, SB 242 n = 12, SB 206 n = 10. lSol/rSol, left/right soleus muscle. [file Image_2.JPEG]

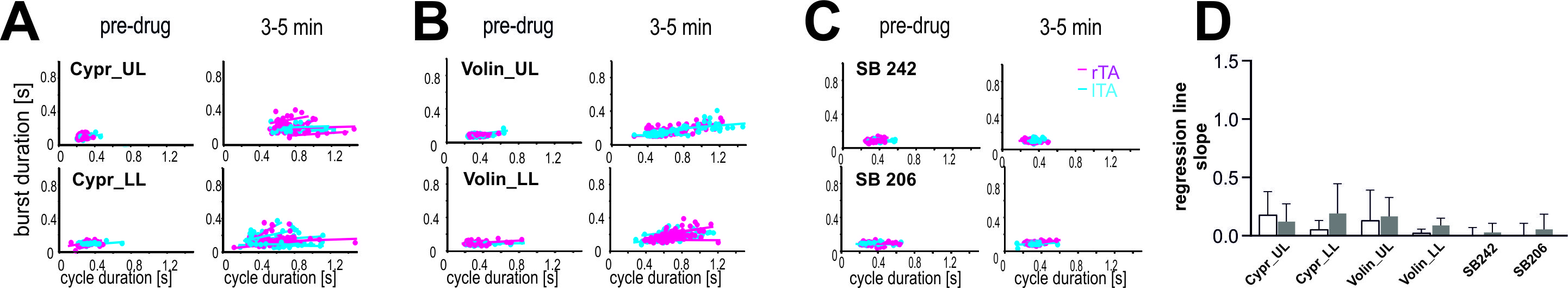

Supplement: FIGURE S3 — The relationship between TA EMG burst duration vs. step cycle duration before and after Cyproheptadine (A), Volinanserin (B) and SB 242084 or SB 206553 (C) applications in the upper lumbar (UL) or low lumbar (LL) spinal cord established using regression line analysis. In the right panel (D) the bars illustrate the mean (±SD) of regression line slopes established for hindlimb muscles in different experimental conditions. The significance of differences in the slopes in various groups of animals before and after various treatment was tested using nonparametric Kruskal–Wallis test followed by Dunn’s test for multiple comparisons (*p < 0.05, **p < 0.01, ***p < 0.001, ****p < 0.0001). Results from left and right muscles were pooled in all the rats in the various experimental conditions, so the number of samples taken for analysis were: Cypr UL n = 12, Cypr LL n = 10, Volin UL n = 10, Volin LL n = 10, SB 242 n = 12, SB 206 n = 10. lTA/rTA, left/right tibialis anterior muscle. [file Image_3.JPEG]

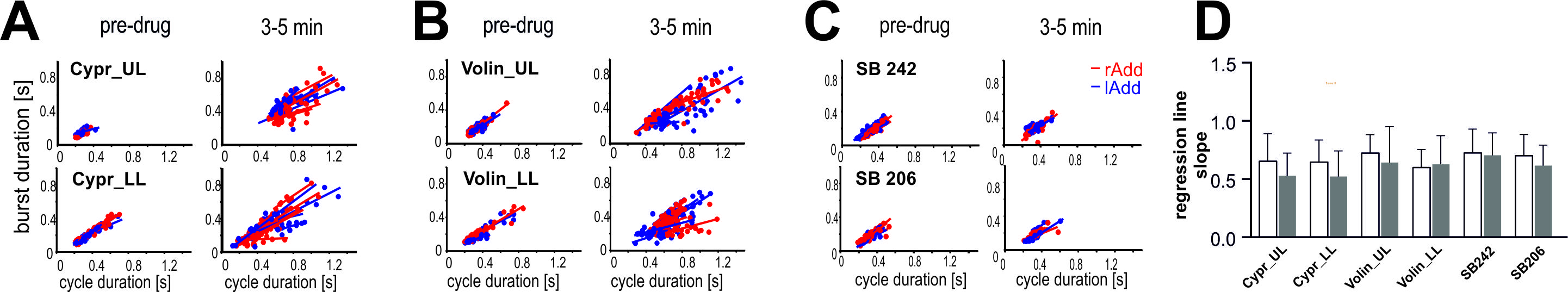

Supplement: FIGURE S4 — The relationship between Add EMG burst duration vs. step cycle duration before and after Cyproheptadine (A), Volinanserin (B) and SB 242084 or SB 206553 (C) applications in the upper lumbar (UL) or low lumbar (LL) spinal cord established using regression line analysis. In the right panel (D) the bars illustrate the mean (±SD) of regression line slopes established for hindlimb muscles in different experimental conditions. The significance of differences in the slopes in various groups of animals before and after various treatment was tested using nonparametric Kruskal–Wallis test followed by Dunn’s test for multiple comparisons (*p < 0.05, **p < 0.01, ***p < 0.001, ****p < 0.0001). Results from left and right muscles were pooled in all the rats in the various experimental conditions, so the number of samples taken for analysis were: Cypr UL n = 12, Cypr LL n = 10, Volin UL n = 10, Volin LL n = 10, SB 242 n = 12, SB 206 n = 10. lAdd/rAdd, left/right adductor muscle. [file Image_4.JPEG]
